# Community detection with node attributes in multilayer networks

**DOI:** 10.1038/s41598-020-72626-y

**Published:** 2020-09-25

**Authors:** Martina Contisciani, Eleanor A. Power, Caterina De Bacco

**Affiliations:** 1 Max Planck Institute for Intelligent Systems, Cyber Valley, 72076 Tübingen, Germany; 2 Department of Methodology, London School of Economics and Political Science, London, WC2A 2AE UK

**Keywords:** Applied mathematics, Computer science, Statistics

## Abstract

Community detection in networks is commonly performed using information about interactions between nodes. Recent advances have been made to incorporate multiple types of interactions, thus generalizing standard methods to multilayer networks. Often, though, one can access additional information regarding individual nodes, attributes, or covariates. A relevant question is thus how to properly incorporate this extra information in such frameworks. Here we develop a method that incorporates both the topology of interactions and node attributes to extract communities in multilayer networks. We propose a principled probabilistic method that does not assume any a priori correlation structure between attributes and communities but rather infers this from data. This leads to an efficient algorithmic implementation that exploits the sparsity of the dataset and can be used to perform several inference tasks; we provide an open-source implementation of the code online. We demonstrate our method on both synthetic and real-world data and compare performance with methods that do not use any attribute information. We find that including node information helps in predicting missing links or attributes. It also leads to more interpretable community structures and allows the quantification of the impact of the node attributes given in input.

## Introduction

Community detection is a fundamental task when investigating network data. Its goal is to cluster nodes into communities and thus find large-scale patterns hidden behind interactions between many individual elements.

The range of application of this problem spans several disciplines. For instance, community detection has been used in sociology to analyze terrorist groups in online social networks^1^; in finance to detect fraud events in telecommunication networks^2^; in engineering to refactor software packages in complex software networks^3^; and in biology to investigate lung cancer^4^ and to explore epidemic spreading processes^5^. In recent years, the variety of fields interested in this topic has broadened and the availability of rich datasets is increasing accordingly. However, most research approaches use only the information about interactions among nodes, in other words the network topology structure. This information can be complex and rich, as is the case for multilayer networks where one observes different types of interactions. For instance, in social networks, interactions could entail exchanging goods, socializing, giving advice, or requesting assistance. Most network datasets, however, contain additional information about individuals, attributes which describe their features, for instance their religion, age, or ethnicity. Node attributes are often neglected *a priori* by state-of-the-art community detection methods, in particular for multilayer networks. They are instead commonly used *a posteriori*, acting as candidates for “ground-truth” for real-world networks to measure the quality of the inferred partition^6,7^, a practice that can also lead to incorrect scientific conclusions^8^. It is thus a fundamental question how to incorporate node attributes into community detection in a principled way. This is a challenging task because one has to combine two types of information^9^, while evaluating the extent to which topological and attribute information contribute to the network’s partition^10^.

To tackle these questions, we develop MTCOV, a mathematically rigorous and flexible model to address this problem for the general case of multilayer networks, i.e., in the presence of different types of interactions. The novelty of this model relies on a principled combination of the multilayer structure together with node information to perform community detection. To the best of our knowledge, MTCOV is the first overlapping community detection method proposed for multilayer networks with node attributes. The model leverages two sources of information, the topological network structure and node covariates (or attributes), to partition nodes into communities. It is flexible as it can be applied to a variety of network datasets, whether directed, weighted, or multilayer, and it outputs overlapping communities, i.e., nodes can belong to multiple groups simultaneously. In addition, the model does not assume any *a priori* correlation structure between the attributes and the communities. On the contrary, the contribution of the attribute information is quantitatively given as an output of the algorithm by fitting the observed data. The magnitude of this contribution can vary based on the dataset. Even if this is not very high (for instance if the attributes are noisy or sparse) the model is nevertheless able to use this extra information to improve performance. At the same time, if incorporating attribute information hurts inference tasks, the model will downweigh this contribution and instead use mostly the topological network structure.

Our method allows domain experts to investigate particular attributes and select relevant community partitions based on what type of node information they are interested in studying. In fact, by choosing the input data, we can drive the algorithm to select for communities that are more relevant to the attribute under study. If the attribute hurts performance and is consequently downweighted by the algorithm, this can be used as a signal that the attribute might not correlate well with any partition, given the remaining topological information available, and thus inform the expert accordingly.

We study MTCOV on synthetic multilayer networks, a variety of single-layer node-attributed real networks and several real multilayer networks of social support interactions in two Indian villages. We measure performance based on prediction tasks and overlap with ground-truth (when this is known). For single-layer networks, we compare the performance of MTCOV to state-of-the-art community detection algorithms with node attributes; for multilayer networks, we test against a state-of-the-art algorithm that does not use any node attribute information and measure the extent to which knowing both types of information helps inference. We find that MTCOV performs well in predicting missing links and attributes. It also leads to more interpretable community structures and allows the quantification of the impact of the node attributes given as input.

To summarize, we present MTCOV, a new method that incorporates both the topology of interactions and node attributes to extract communities in multilayer networks. It is flexible, efficient and it has the property of quantitatively estimating the contributions of the two sources of information. It helps domain experts to investigate particular attributes and to better interpret the resulting communities. Moreover, by including relevant node attributes, it boosts performance in terms of edge prediction.

### Related work

Several methods have been proposed to study community detection in networks^11^. In particular, we are interested in those valid for multilayer networks^12^. These generalize single-layer networks in that they can model different types of interactions and thus incorporate extra information that is increasingly available. Among these, we focus on generative models for multilayer networks^13–19^, which are based on probabilistic modeling like Bayesian inference or maximum likelihood optimization. These are flexible and powerful in terms of allowing multiple inference tasks, injecting domain knowledge into the theoretical framework, and being computationally efficient. However, the majority of these methods do not consider node attributes as input along with the network information. In fact, the few methods developed for community detection in multilayer networks with node attributes are based on first aggregating the multilayer network into a single layer, either by combining directly the adjacency matrices of each layer^20^ or by using similarity matrices derived from them along with the node attributes^21,22^. In the context of data mining, a similar problem can be framed for learning low dimensional representations of heterogeneous data with both content and linkage structure (what we call attributes and edges). This is tackled by using embeddings extracted via deep architectures^23^, which is rather different than our approach based on statistical inference. Our problem bears some common ground with the one studied by Sachan et al.^24^ for extracting communities in online social networks, where users gather based on common interests; they adopt a Bayesian approach, but with a rather different goal of associating different types of edges to topics of interest. A related but different problem is that of performing community detection with node attributes on multiple independent networks^25,26^; this differs with modeling a single multilayer network in that it assumes that covariates influence in the same way all the nodes in a network but in a different way the various networks in the ensemble. For single-layer networks, there has been more extensive work recently on incorporating extra information on nodes^9,25,27–34^. Among those adopting probabilistic modeling, some incorporate covariate information into the prior information of the latent membership parameters^25,35,36^, while others include covariates in an additive way along with the latent parameters^37,38^, so that covariates influences the probability of interactions independently of the latent membership.

These works show the impact of adding nodes attributes in community detection *a priori* into the models to uncover meaningful patterns. One might then be tempted to adopt such methods also in multilayer networks by collapsing the topological structure into a suitable single network that can then be given in input to these single-layer and node-attributed methods as done by Gheche et al.^20^. However, collapsing a multilayer network often leads to important loss of information, and one needs to be careful in determining when this collapse is appropriate and how it should be implemented, as shown for community detection methods without attribute information^39,40^. Thus the need of a method that not only incorporates different types of edges but also node attributes.

## Results

We test MTCOV’s ability to detect communities in multilayer networks with node attributes by considering both synthetic and real-world datasets. We compare against MULTITENSOR^13^, an algorithm similar to ours but that does not include node attributes. We also test MTCOV’s performance on single-layer networks, as the mathematical framework behind MTCOV still applies. Given this potential use and the paucity of algorithms suitable for comparison for multilayer networks, such comparisons assess the general utility of MTCOV.

### Multilayer synthetic networks with ground-truth

**Figure 1:**
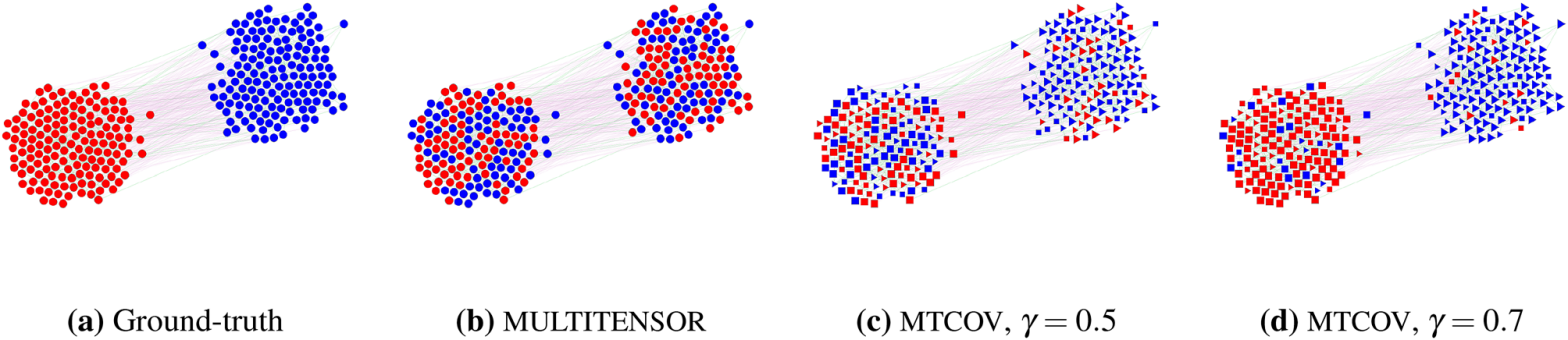
Partition of a synthetic multilayer network with attributes. We generated synthetic directed multilayer networks using a stochastic block model, that aligns with $G_1$. To illustrate, here we do the equivalent task on a smaller network of size N = 299, C = 2 communities of equal-size unmixed group membership and L = 2 layers, of which one is assortative (green) and one disassortative (pink); (**a**) the ground-truth partition; (**b**–**d**) the communities inferred by three different methods: (**b**) MULTITENSOR, an algorithm without attributes, (**c**) MTCOV using the network structure and the attributes with the same proportion, i. e. $\gamma =0.5$ and (**d**) MTCOV using mostly the attribute structure, i.e. $\gamma =0.7$. Colors denote the inferred partition; the attributes in (**c**) and (**d**) are generated by matching them with true community assignments for the $50\%$ and $70\%$ of the nodes respectively, and chosen uniformly at random from the non-matching values; square and triangle denote the synthetic dummy attribute (squares are matched with the red group, triangles with the blue) and the size of the node shows the nodes matched with the true community (bigger means deterministic match, smaller means uniform at random match). We use the matrix *U* for the membership.

To illustrate the flexibility and the robustness of our method, we generate multilayer synthetic networks with different kinds of structures in the various layers adapting the protocol described in De Bacco et al.^13^ to accommodate node attributes. We generate attributes as done in Newman and Clauset^27^: we match them with planted communities in increasing ratios varying from 0.3 to 0.9; these values correspond also to the $\gamma$ parameters that we fix for MTCOV. Specifically, we generate three types of directed networks using a stochastic block model^41^, all with $C=2$ communities of equal-size unmixed group membership and $N=1000$ nodes, but with different numbers and kinds of layers, similar to De Bacco et al.^13^. The first network ($G_{1}$) has $L=2$ layers, one assortative ($W^{\alpha }$ has higher diagonal entries) and one disassortative ($W^{\alpha }$ has higher off-diagonal entries); the second ($G_{2}$) has $L=4$ layers, two assortative and two disassortative and the third ($G_{3}$) has $L=4$ layers, one assortative, one disassortative, one core-periphery ($W^{\alpha }$ has higher diagonal entries but one of the two is bigger than the other) and one with biased directed structure ($W^{\alpha }$ has higher off-diagonal entries but one of the two is bigger than the other). We generate ten independent samples of each of these types of networks and use all the evaluation metrics described in the “Methods” section in the presence of ground-truth. We use the membership inferred by the algorithms using the best maximum likelihood fixed point over 10 runs with different random initial conditions. As shown in Table 1, MTCOV performs significantly better than MULTITENSOR on the first and second network. This suggests that incorporating attribute information can significantly boost inference, with an increasing benefit for a smaller number of layers. Figure 1 shows an example of this result. Notice that $G_{2}$ requires a smaller match ($\gamma =0.5)$ between attributes and communities than $G_{1}$ ($\gamma =0.7)$ to achieve similar performance. $G_{1}$ and $G_{2}$ have similar structure, but the second has twice as many layers. Thus, increasing the number of layers may require less contribution from the extra information of the attributes, a possible advantage for multilayer networks. This intuition is reinforced by noticing not only that the best performance is achieved for $\gamma <0.9$, but also that both the algorithms perform very well in the third network, regardless of the value of the match between attributes and communities. Contrary to $G_{2}$, $G_{3}$ has a different structure in each of the 4 layers. This diversity can be even more beneficial than having more but correlated layers (as in $G_{1}$ vs. $G_{2}$). These synthetic tests demonstrate the impact of leveraging both node attributes and topological information: when topological structure is not very informative (as in $G_{1}$ with only two layers), adding node attributes can significantly help in recovering the communities. In contrast, when topological information is more complex (as in $G_{3}$ where all layers are different), properly combining the different layers’ structures can compensate for a limited access to extra information on nodes. Overall, this shows the need for methods suited for exploiting various sources of information and the complexity behind multilayer networks.

**Table 1:** Performance of algorithms MULTITENSOR and MTCOV on synthetic multilayer networks with attributes.

Method	$G_{1}$				$G_{2}$				$G_{3}$			
	F1-score	Jaccard	CS	$\hbox {L}_1$	F1-score	Jaccard	CS	$\hbox {L}_1$	F1-score	Jaccard	CS	$\hbox {L}_1$
MULTITENSOR	$0.512\pm 0.006$	$0.344\pm 0.006$	$0.585\pm 0.005$	$0.492\pm 0.004$	$0.514\pm 0.006$	$0.346\pm 0.06$	$0.614\pm 0.005$	$0.490\pm 0.005$	$\mathbf{0.999 }\pm \mathbf{0.001 }$	$\mathbf{0.998 }\pm \mathbf{0.001 }$	$\mathbf{0.991 }\pm \mathbf{0.001 }$	$0.063\pm 0.002$
MTCOV_0.3	$0.7\pm 0.2$	$0.5\pm 0.2$	$0.7\pm 0.1$	$0.4\pm 0.1$	$0.8\pm 0.2$	$0.7\pm 0.2$	$0.8\pm 0.1$	$0.3\pm 0.2$	$0.995\pm 0.002$	$0.990\pm 0.004$	$0.984\pm 0.002$	$0.080\pm 0.004$
MTCOV_0.5	$0.6\pm 0.1$	$0.5\pm 0.2$	$0.7\pm 0.1$	$0.4\pm 0.1$	$0.992\pm 0.005$	$0.985\pm 0.009$	$0.986\pm 0.004$	$0.064\pm 0.004$	$0.996\pm 0.002$	$0.992\pm 0.004$	$0.985\pm 0.002$	$0.079\pm 0.004$
MTCOV_0.7	$\mathbf{0.988 }\pm \mathbf{0.002 }$	$\mathbf{0.976 }\pm \mathbf{0.004 }$	$\mathbf{0.977 }\pm \mathbf{0.002 }$	$0.079\pm 0.003$	$\mathbf{1. }\pm \mathbf{0. }$	$\mathbf{1.000 }\pm \mathbf{0.001 }$	$\mathbf{0.991 }\pm \mathbf{0.001 }$	$0.062\pm 0.002$	$0.994\pm 0.002$	$0.988\pm 0.004$	$0.982\pm 0.001$	$0.087\pm 0.002$
MTCOV_0.9	$0.958\pm 0.003$	$0.920\pm 0.005$	$\mathbf{0.977 }\pm \mathbf{0.001 }$	$\mathbf{0.050 }\pm \mathbf{0.002 }$	$0.992\pm 0.002$	$0.984\pm 0.004$	$0.988\pm 0.001$	$\mathbf{0.050 }\pm \mathbf{0.002 }$	$0.976\pm 0.003$	$0.952\pm 0.006$	$0.982\pm 0.002$	$\mathbf{0.051 }\pm \mathbf{0.003 }$

We use different matches (one per row, e.g., ${{\text{MTCOV}}}\_{0.3}$ denotes a match of 0.3, this is also the value we use to fix $\gamma$) between attributes and planted communities on synthetic directed multilayer networks. Results are averages and standard deviations over 10 networks samples for each network type $G_{m}$, $m=1,2,3$; we take the average performance over the incoming and outgoing memberships, i.e., the matrices *U* and *V*, and the best performances are in boldface. Networks are generated with stochastic block model with $C=2$, $N=1000$ and average degree $k=4$. Denote $W^{a}$, $W^{d}$, $W^{cp}$ and $W^{bd}$, the affinity matrices of the assortative, disassortative, core-periphery and the biased directed layers respectively. Then, their entries are $w^{a}_{11}=w^{a}_{22}=w^{d}_{12}=w^{d}_{21}=w^{cp}_{11}=w^{bd}_{12}=\frac{kC}{N}$, $w^{a}_{12}=w^{a}_{21}=w^{d}_{11}=w^{d}_{22}=w^{cp}_{12}=w^{cp}_{21}=w^{bd}_{11}=w^{bd}_{22}=0.1\times \frac{kC}{N}$ and $w^{cp}_{22}=w^{bd}_{12}=0.03\times \frac{kC}{N}$. The F1-score, Jaccard, CS and $\hbox {L}_1$ are performance metrics as defined in the “Methods” section.

### Multilayer social support network of rural Indian villages

We demonstrate our model beyond synthetic data by applying it to social support networks of two villages in Tamil Nadu, India, which we call by the pseudonyms “Tenpaṭṭi” (Ten) and “Alakāpuram” (Ala)^42–44^. Data were collected in the form of surveys where adult residents were asked to nominate those individuals who provided them with various types of support, including running errands, giving advice, and lending cash or other household items. These were collected in two rounds, one in 2013 and the other in 2017. Each type of support corresponds to a layer in the network; we consider only those layers present in both rounds, for a total of $L=6$ layers. After pre-processing the data, by considering only those individuals who had at least one outgoing edge and removing self-loops, the resulting networks have the size reported in Table 2. In addition, several attributes were collected, which include information about age, religion, caste, and education level. Ethnographic observation in these villages^42^ and previous analyses^43,44^ suggest that social relations are strongly structured by religious and caste identity, with these divisions shaping where people live, who they marry, and who they choose to associate with. In other words, they suggest a dependence between the attributes Religion and Caste and the mechanisms driving edge formation in these social support networks. Motivated by these insights, here we consider the attributes Caste and Religion and add them into the model. In addition, we test the importance of variables that we expect to be less informative, such as gender and age. The latter, being continuous, is also an example of a non-categorical variable. Provided it has a finite range, as it is the case for age, we can encode it into categorical by binning its values. Here we use equal bins of size 5 years.

**Table 2:** Network summary statistics for the four social support networks of Indian villages.

Village	Year	Nodes	Edges	${\langle k \rangle }$	Caste	Religion	Age	Gender
Alakāpuram	2013	419	4,161	20	14	3	11	2
	2017	441	5,578	25	13	3	12	2
Tenpaṭṭi	2013	362	3,374	19	11	2	11	2
	2017	346	3,806	22	9	2	12	2

Each has the same set of 6 layers and Edges are the total over them; $\langle k \rangle$ is the average degree per node on the whole multilayer network. The columns Caste, Religion, Age and Gender are the number of different categories observed in each network for their respective attribute.

Without assuming *a priori* any ground-truth, we measure performance using the AUC and accuracy as explained in the “Methods” section. We compare with MULTITENSOR to measure the extent to which adding the attributes helps predicting edges and attributes; in addition, in terms of accuracy values, we consider two baselines for further comparisons: (1) a uniform at random probability over the number of possible categories (RP); and (2) the maximum relative frequency of the attribute value appearing more often (MRF). We fix hyperparameters using 5-fold cross-validation along with grid-search procedure (see “Cross-validation tests and hyperparameter settings” section for more details). We obtain values of $\gamma \in [0.2, 0.9]$, signalling relevant correlations between attributes and communities. For details, see Supplementary Table S2. Empirically, we observe that when $\gamma >0.5$ the algorithm achieves better performance in terms of link and attribute prediction by well balancing the log-likelihood of the attribute dimension and the one of the network structure.

**Figure 2:**
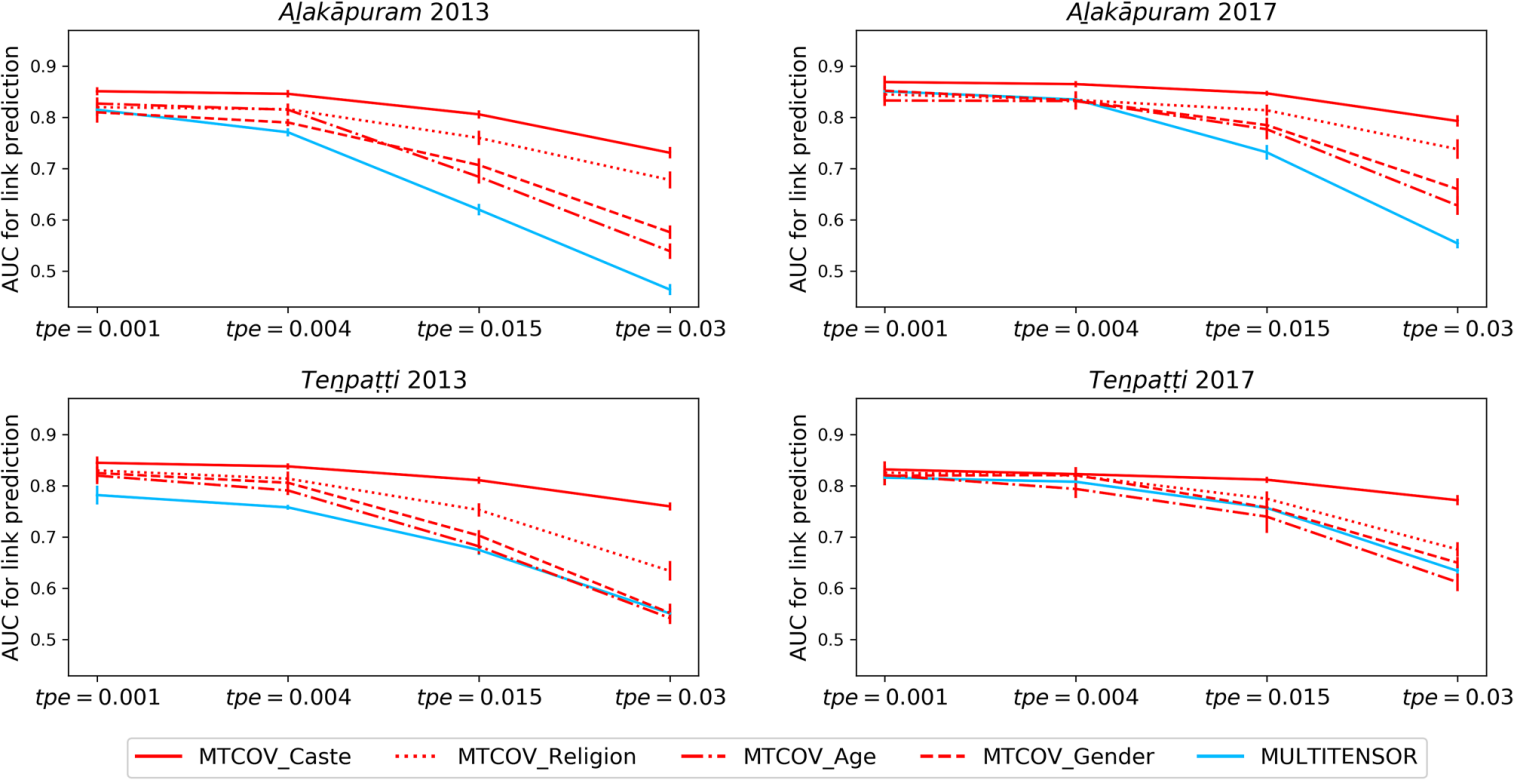
Probabilistic link prediction with biased edge sampling. Results are AUC values of MTCOV and MULTITENSOR on four social support networks in different held-out settings. Here *tpe* indicates the total probability of selecting one edge (positive example) in the test set. We consider Caste, Religion, Age and Gender attributes; results are averages and standard deviations over 10 independent runs.

**Figure 3:**
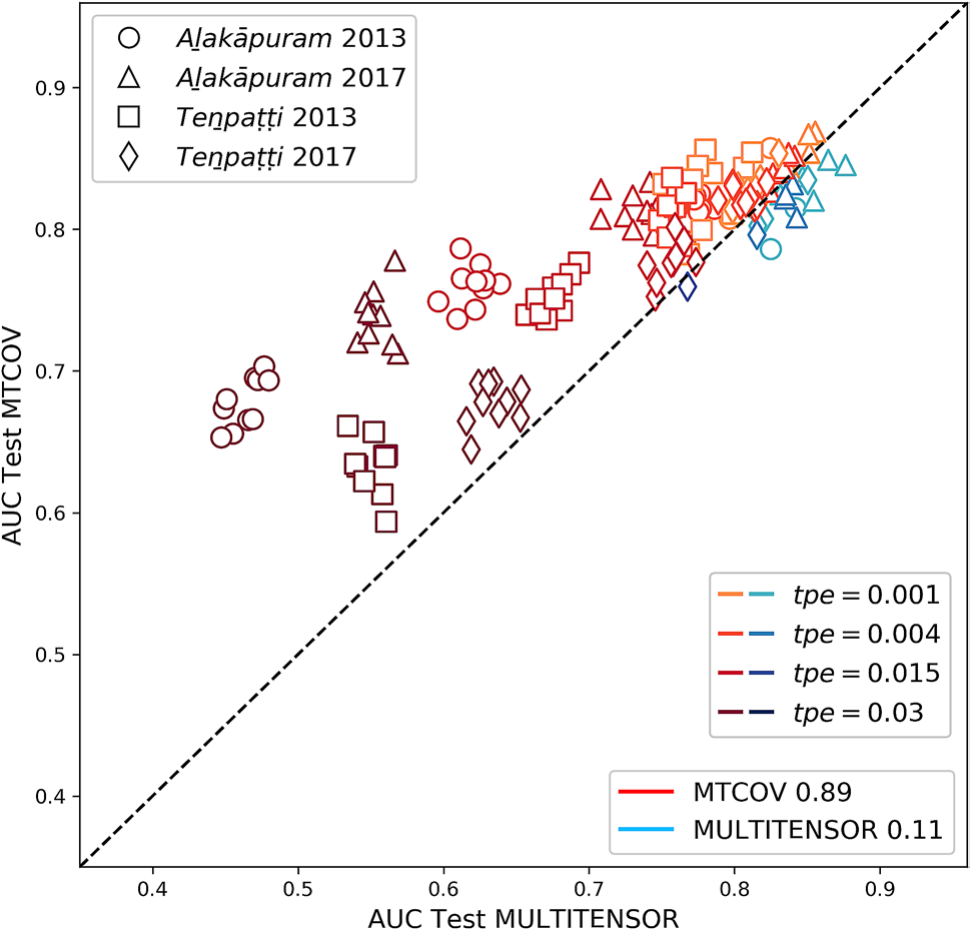
Trial-by-trial probabilistic link prediction with biased edge sampling. The values of AUC for MTCOV and MULTITENSOR are shown on the vertical axis and the horizontal axis respectively. The brightness represents the hardness of the settings in terms of biasing the edge sampling in the training. From bottom to top: $tpe=0.03$ (hard, dark color), $tpe=0.015$, $tpe=0.004$ (random), $tpe=0.001$ (easy, light color). Points above the diagonal, shown in shades of red, are trials where MTCOV is better performing than MULTITENSOR. The fractions for which each method is superior are shown in the plot legend. We use the attribute Religion.

For validation, we split the dataset into training/test sets uniformly at random as explained in the “Methods” section. Table 3 reports the average results over ten runs for each network, and shows that MTCOV is capable of leveraging two sources of information to improve both performance metrics. In fact, our algorithm systematically achieves the highest accuracy for attribute prediction and the highest AUC for edge prediction (boldface). While a good performance in attribute prediction is expected by design as we add this data into the model, the fact that it also boosts performance in terms of edge prediction is not granted *a priori*. Instead, it is a quantitative way to show that an attribute plays an important role in the system. It also demonstrates the potential of capturing correlations between two different sources of information, which can have relevant applications, in particular when missing information of one kind. Notice in particular the improvement in AUC when using caste compared to no attribute given (MULTITENSOR). The other attributes are less informative; in particular age has a performance similar to MULTITENSOR in edge prediction, signalling that it does not contribute to inform edge formation. Indeed, it has the smallest inferred $\gamma$ (always $< 0.5$), which gives also worse accuracy performance than the baseline, signalling again that this attribute may not be correlated with the community structure. All these results show the flexibility of MTCOV in adapting based on the data given in input: if warranted, it is able to ignore those attributes that are not correlated with network division and instead find communities that are mainly based on the network structure. Next, we test how adding node attributes impacts robustness against unbalanced data, where the ratio of positive examples (existing edges) observed in the training is different than that in the test set. We denote the total probability of selecting an edge in the test as *tpe* and consider values $\textit{tpe} \in \left\{ 0.001,\, 0.004,\, 0.015, \, 0.03\right\}$ denoting under-representation (0.001), equal (0.004), and over-representation (values 0.015 and 0.03) compared to the uniform at random selection (empirically we find $tpe=0.004$). In these tests, we hold out $20\%$ of the entries of *A* biasing their selection using the *tpe* values; in addition, we give as input the whole design matrix *X* (attributes) and measure link prediction performance. We observe that MTCOV is significantly more robust than the algorithm that does not use any attribute information, regardless of the value of $\gamma$. In fact, even though performance deteriorates as we decrease the number of positive examples in the training set (i.e., higher *tpe*), MTCOV is less impacted by this, as shown in Fig. 2 (results reported in Supplementary Table S3). Notice in particular performance discrepancies when using the attribute Caste in the difficult regimes ($\textit{tpe} \in \left\{ 0.015, 0.03\right\}$): MTCOV’s performance deteriorates only a little, while using the other attributes or no attribute makes performance significantly worse, with AUC down to 0.6 from a value higher than 0.8 for easier regimes. Moreover, notice that attributes with the same scaling parameter value can give different prediction results, underlying the necessity to consider both the value of the estimated $\gamma$ and the quality of the attribute to quantify its importance. This could explain why Caste provides always better results, given by the fact that its categories are more heterogeneous (i.e., more information) than Religion and Gender. The robustness of MTCOV is also confirmed by analyzing the performances on a trial-by-trail basis, each trial being a random sample of the held-out entries. As we show in Fig. 3, MTCOV better predicts links in $89\%$ of the trials and never goes below the threshold of 0.5, the baseline random choice. These results demonstrate how adding another source of information helps when observing a limited amount of network edges.

**Table 3:** Prediction performance on real multilayer networks with attributes.

Attribute	Method	ACCURACY for attribute prediction				AUC for link prediction			
		Ala 2013	Ala 2017	Ten 2013	Ten 2017	Ala 2013	Ala 2017	Ten 2013	Ten 2017
	MULTITENSOR					$0.771\pm 0.009$	$0.835\pm 0.006$	$0.758\pm 0.005$	$0.81\pm 0.01$
Caste	RP	0.07	0.08	0.10	0.11				
	MRF	$0.556\pm 0.009$	$0.57\pm 0.01$	$0.32\pm 0.01$	$0.33\pm 0.02$				
	MTCOV	$\mathbf{0.80 }\pm \mathbf{0.05 }$	$\mathbf{0.77 }\pm \mathbf{0.05 }$	$\mathbf{0.69 }\pm \mathbf{0.09 }$	$\mathbf{0.74 }\pm \mathbf{0.07 }$	$\mathbf{0.837 }\pm \mathbf{0.009 }$	$\mathbf{0.858 }\pm \mathbf{0.008 }$	$\mathbf{0.829 }\pm \mathbf{0.006 }$	$\mathbf{0.82 }\pm \mathbf{0.01 }$
Religion	RP	0.33	0.33	0.50	0.50				
	MRF	$0.837\pm 0.008$	$0.843\pm 0.006$	$0.696\pm 0.008$	$0.679\pm 0.008$				
	MTCOV	$\mathbf{0.96 }\pm \mathbf{0.02 }$	$\mathbf{0.95 }\pm \mathbf{0.03 }$	$\mathbf{0.76 }\pm \mathbf{0.08 }$	$\mathbf{0.80 }\pm \mathbf{0.05 }$	$0.813\pm 0.007$	$0.83\pm 0.01$	$0.81\pm 0.02$	$0.80\pm 0.01$
Age	RP	0.09	0.08	0.09	0.08				
	MRF	$\mathbf{0.135 }\pm \mathbf{0.005 }$	$\mathbf{0.126 }\pm \mathbf{0.007 }$	$0.126\pm 0.005$	$\mathbf{0.128 }\pm \mathbf{0.008 }$				
	MTCOV	$0.11\pm 0.03$	$0.11\pm 0.02$	$\mathbf{0.13 }\pm \mathbf{0.04 }$	$0.10\pm 0.03$	$0.80\pm 0.01$	$0.823\pm 0.008$	$0.783\pm 0.009$	$0.80\pm 0.01$
Gender	RP	0.50	0.50	0.50	0.50				
	MRF	$0.584\pm 0.009$	$0.58\pm 0.01$	$0.56\pm 0.01$	$0.55\pm 0.01$				
	MTCOV	$\mathbf{0.61 }\pm \mathbf{0.05 }$	$\mathbf{0.65 }\pm \mathbf{0.04 }$	$\mathbf{0.58 }\pm \mathbf{0.08 }$	$\mathbf{0.71 }\pm \mathbf{0.08 }$	$0.79\pm 0.02$	$0.831\pm 0.009$	$0.80\pm 0.01$	$0.81\pm 0.01$

Results are averages and standard deviations over 10 independent trials of cross-validation with 80–20 splits selected uniformly at random (i.e., $\textit{tpe}=0.004$); the best performances are in boldface. Datasets are described in Table 2. RP is the performance of uniform random probability and MRF the one of the maximum relative frequency, see “Methods” section for details.

#### Qualitative analysis of a social support network

**Figure 4:**
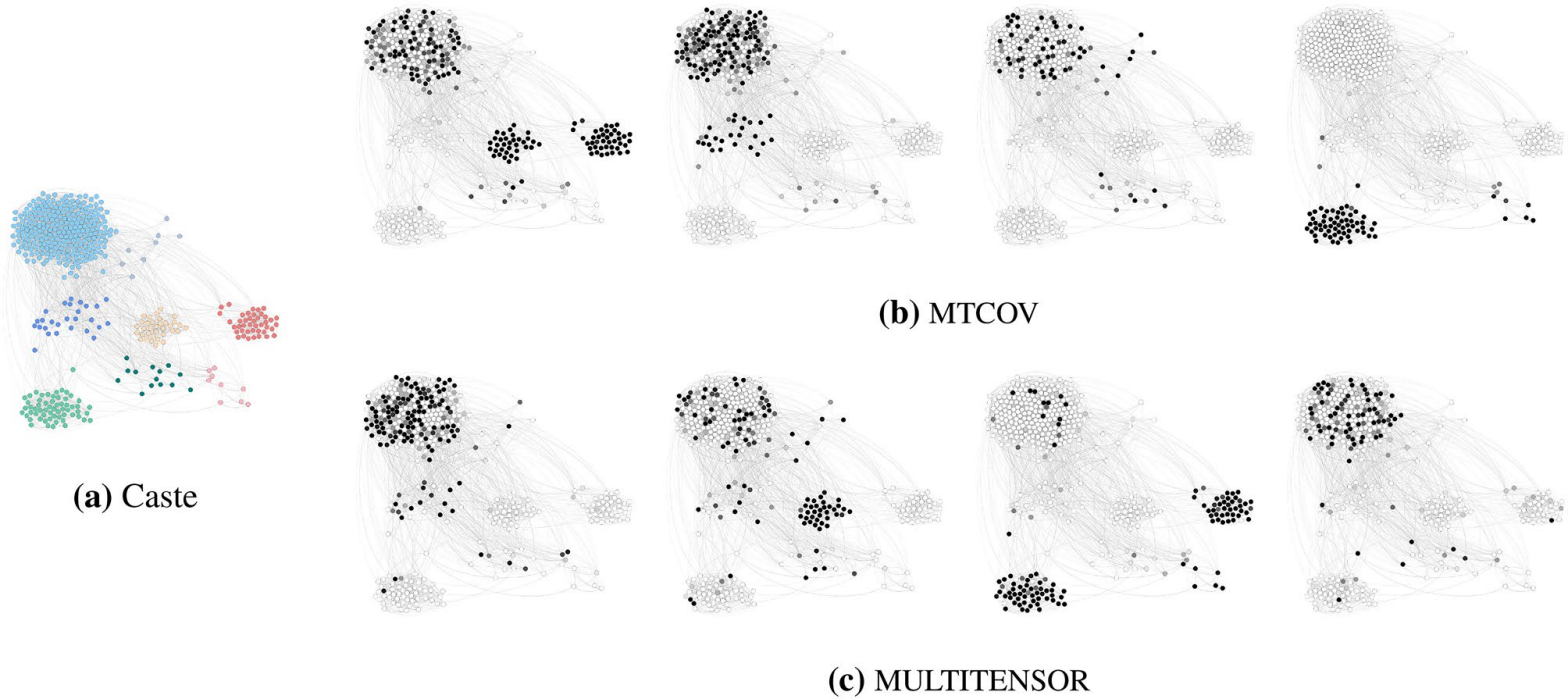
Attributes and inferred communities. Nodes of the social support network of Alakāpuram in 2017 are colored by: (**a**) the attribute Caste (with colors as shown in Fig. 5); inferred communities by (**b**) MTCOV and (**c**) MULTITENSOR. Darker values in the grey scales indicate higher values of the entry of the membership vector $u_i$.

**Figure 5:**
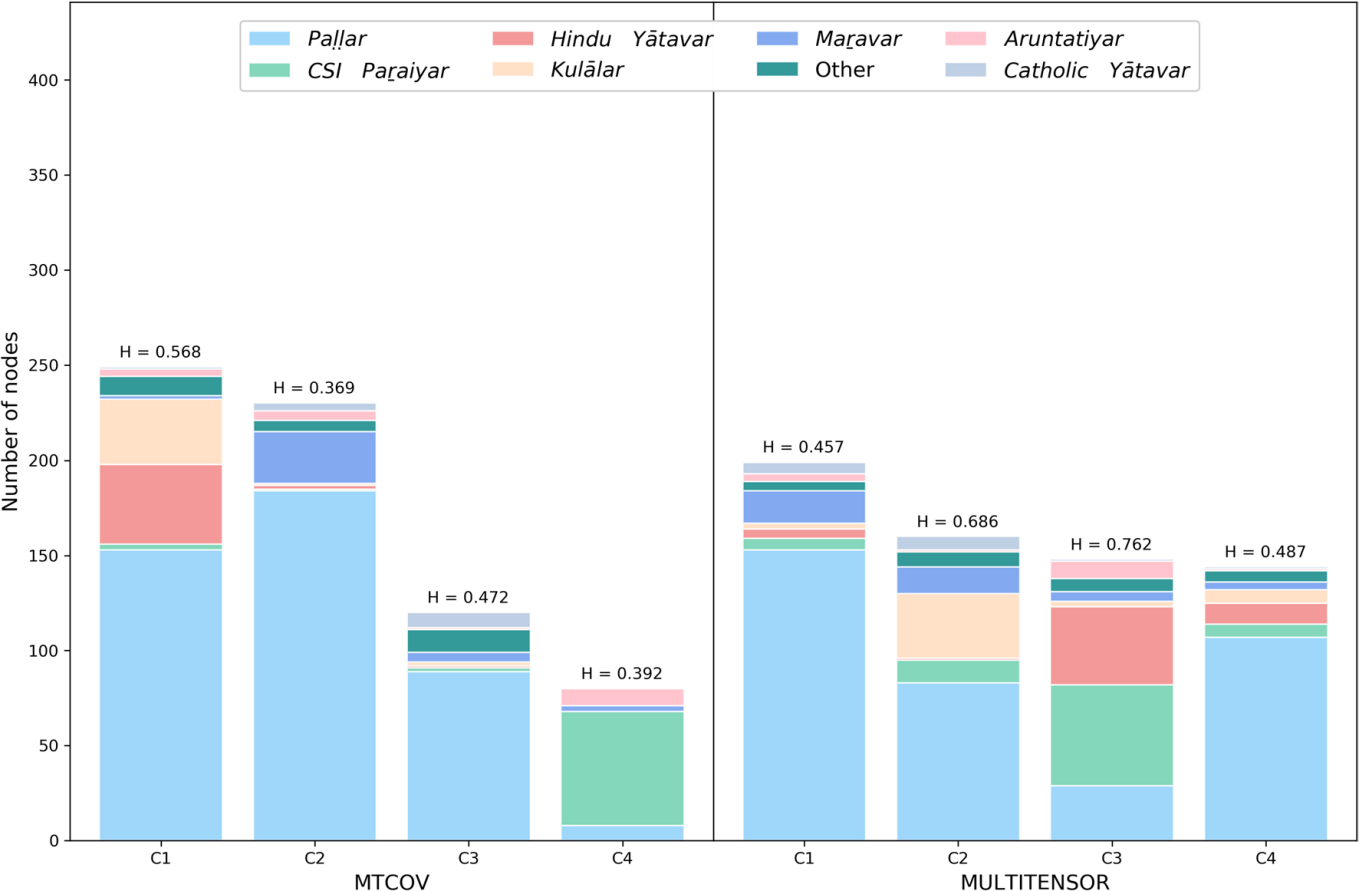
Partition of the attribute Caste inside each community detected by MTCOV and MULTITENSOR in the social support network of Alakāpuram in 2017. The category Other contains small categories having less than five individuals. The label on top of each bar is the value of the entropy of the variable Caste inside the corresponding community. Note that nodes can have mixed membership, here we build a group *k* by adding to it all nodes *i* that have a non-zero *k*-th entry $u_{ik}$. The number of nodes is $N=441$, corresponding to the maximum value of the y-axis plotted.

To demonstrate our MTCOV model beyond prediction tasks and highlight its potential for interpretability, we show as an example its qualitative behavior on the real network of Alakāpuram in 2017 (see Table 2). Specifically, we compare the communities extracted by our algorithm and those inferred by MULTITENSOR. To ease comparison, we fix the same number of groups to $C=4$ for both algorithms and measure how caste membership distributes across communities, and fix $\gamma =0.8$ as obtained with cross-validation. Figure 4 shows the magnitude of each individual’s inferred outgoing memberships $u_{i}$ in each group. While the communities identified by MTCOV and MULTITENSOR show substantial similarities, MTCOV generally classifies castes more consistently into distinct communities, as we show in Figs. 4 and 5. To make a quantitative estimate of the different behaviors, we measure the entropy of the attribute inside each community $H_{k}=-\sum _{z=1}^{Z}f_{z}\log f_{z}/\log (Z)$, where $f_{z}$ is the relative frequency of the *z*-th caste inside a group *k*, and the denominator is the entropy of a uniform distribution over the *Z* castes, our baseline for comparison. Values of $H_{k}$ close to 1 denote a more uniform distribution of castes, whereas smaller values denote an unbalanced distribution with most of the people belonging to a few castes. We find that MTCOV has smaller entropies over the groups, with two groups having the smallest values, whereas MULTITENSOR has the highest, showing its tendency to cluster individuals of different castes into the same group. In addition, we observe that MTCOV has a more heterogenous group size distribution which seems to be correlated with caste. Notably, the algorithms differ in how they place two caste groups that live in hamlets separated from the main village (the Hindu Yātavars and CSI Paraiyars). With MULTITENSOR, they are grouped together, while with MTCOV, the Hindu Yātavars are joined up into a community with Paḷḷars and Kulālars. While MULTITENSOR is clearly picking up the structural similarities of the two hamlets, this division makes little sense socially and culturally. In contrast, the way in which MTCOV defines a community which spans caste boundaries (MTCOV C1) aligns with ethnographic knowledge of the relations between these castes. Finally, we remark that there might be multiple meaningful community divisions in the network, and the fact that MTCOV’s partition seems to better capture the distributions in the attribute caste does not mean than one algorithm is better than the other. In fact, there might be other hidden topological properties that MULTITENSOR’s partition is picking up by being agnostic to caste membership. The choice of which algorithm to use should be made based on the final goal of the application at hand.

### Results on single-layer networks

**Figure 6:**
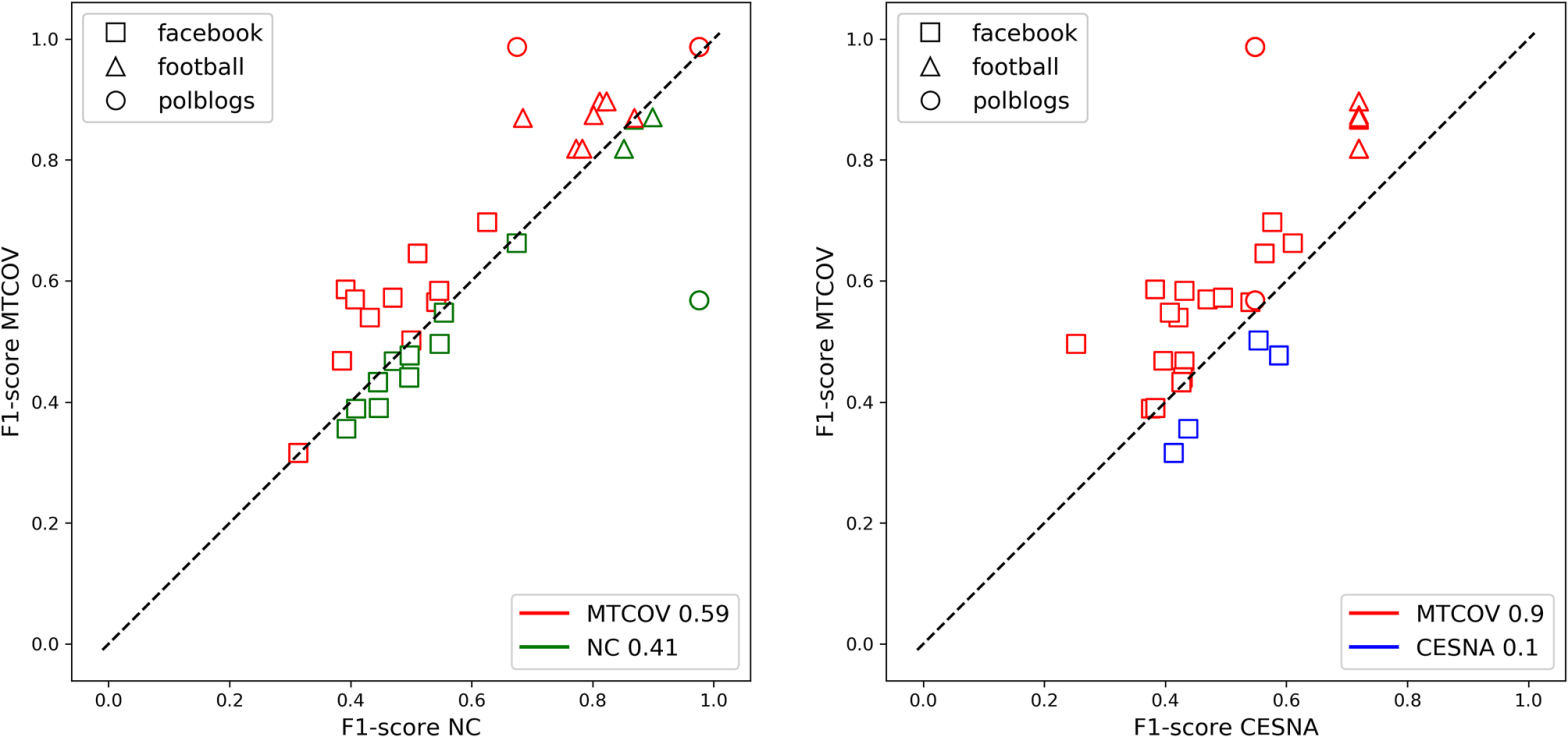
Trial-by-trial performance in F1-score. We compare MTCOV on the y-axis, with on the x-axis (left) NC and (right) CESNA. Markers denote the datasets: squares for *facebook*, triangles for *football* and circles for *polblogs*. Points above the diagonal, shown in red, are trials where MTCOV is more accurate than the other. The fractions for which each method is superior are shown in the plot legend.

Our model can be used for single-layer networks as well. For these we can compare against two state-of-the-art algorithms, both probabilistic generative models but different in their assumptions: CESNA^9^ which considers overlapping communities and posits two independent Bernoulli distributions for network edges and node attributes; and the model proposed by Newman and Clauset^27^ (NC) for non-overlapping communities, a Bayesian approach where the priors on the community memberships depend on the node attributes. CESNA, similarly to our model, assumes conditional independence of the two likelihoods and introduces a regularization parameter between them; it uses block-coordinate ascent for parameters’ estimation, while NC uses an EM algorithm for parameters’ estimation, similarly to what we do here. We test MTCOV against them on both synthetic and real single-layer networks with node attributes, with and without ground-truth. We transform directed networks to undirected because both CESNA and NC do not distinguish for edge directionality. Results on synthetic data show that MTCOV and NC have similar performance in correctly classifying nodes in their ground-truth communities and both are better than CESNA; the main difference is that MTCOV is more stable and has less variance for high attribute correlation, in particular in the hard regime where classification is more difficult. We leave details in the Supplementary Section S4. For single-layer real networks, we use datasets with ground-truth candidates and node attributes: the ego-Facebook network (*facebook*)^45^, a set of 21 networks built from connections between a person’s friends where potential ground-truth are circles of friends hand-labeled by the ego herself; the American College football network (*football*)^46^, a network of football teams playing against each other, where a ground-truth candidate is the conference to which each team belongs; and a network of political blogs (*polblogs*)^47^ where potential ground-truth communities are divided by *left/liberal* and *right/conservative* political parties, see Supplementary Section S4 for details. For each network, we run a 5-fold cross-validation procedure combined with grid-search for fixing the hyperparameter $\gamma$ (see “Cross-validation tests and hyperparameter settings” section for details; note that in this case we use the ground-truth value of *C*, hence $\gamma$ is the only hyperparameter left to be tuned). For *facebook* we find that the average over the 21 networks is $\gamma =0.15$, which signals a low correlation between the covariates and the communities, whereas for the *football* and *polblogs* networks we obtain much higher values of $\gamma$ equal to 0.6 and 0.75 respectively. MTCOV has better performance in terms of F1-score and Jaccard similarity across the majority of datasets, as shown in Table 4. This is also supported by a trial-by-trial comparison shown in Fig. 6 for F1-score (similar results are obtained for Jaccard), where we find that MTCOV is more accurate in $59\%$ and $90\%$ of the cases than NC and CESNA, respectively.

**Table 4:** Performance of methods MTCOV, NC and CESNA on three datasets, according to two different measures used in the Eq. (16).

Method	F1-score			Jaccard similarity		
	facebook	football	polblogs	facebook	football	polblogs
MTCOV	$\mathbf{0.5 }\pm \mathbf{0.1 }$	$\mathbf{0.86 }\pm \mathbf{0.03 }$	$0.8\pm 0.2$	$\mathbf{0.4 }\pm \mathbf{0.1 }$	$\mathbf{0.82 }\pm \mathbf{0.04 }$	$0.8\pm 0.2$
NC	$0.48\pm 0.08$	$0.82\pm 0.06$	$\mathbf{0.95 }\pm \mathbf{0.09 }$	$0.36\pm 0.08$	$0.75\pm 0.08$	$\mathbf{0.9 }\pm \mathbf{0.1 }$
CESNA	$0.46\pm 0.09$	$0.7\pm 0.0$	$0.6\pm 0.0$	$0.33\pm 0.08$	$0.6\pm 0.0$	$0.4\pm 0.0$

The results are averages and standard deviations over ten independent runs and the best outcomes are bolded.

## Discussion

We present MTCOV, a generative model that performs overlapping community detection in multilayer networks with node attributes. We show its robustness in adapting to different scenarios, and its flexibility in exploiting the attributes that are more informative while ignoring those that are less correlated with the network communities. Our method is capable of estimating quantitatively the contribution given by the attributes and incorporating them to improve prediction performance both in terms of recovering missing attributes and in terms of link prediction. This allows domain experts to investigate particular attributes and select relevant community partitions based on what type of node information they are interested in investigating. There are valuable possible extensions of this work. One example is to incorporate modeling of more complex data types for the attributes, for instance combinations of discrete and continuous attributes, or other types of extra information, like time-varying network elements, whether the attributes, node, edges or combinations of these. From a technical point of view, when the topological and attribute datasets are very unbalanced in size, this might impact their relative likelihood weight and thus inference. One should then consider automating the process of rescaling them accordingly, as a pre-processing step to be incorporated into the model. Similarly, hyperparameter selection would benefit from an automatized routine when more than one performance metric is considered. The relations between attributes and communities could be transferred across networks to predict missing information when having access to similar but incomplete datasets. We show examples of these here, where we studied two snapshots of the same village networks across time. While we leave these questions for future work, we provide an open source version of the code.

## Methods

We adapt recent ideas from the generative model behind MULTITENSOR^13^, a multilayer mixed-membership model based on a Poisson tensor factorization^48^, to incorporate node attributes in a principled manner. It can take in input directed and undirected networks, allowing different topological structures in each layer, including arbitrarily mixtures of assortative, disassortative and core-periphery structures. We move beyond MULTITENSOR by incorporating node covariates via introducing a proper likelihood term that accounts for this extra information. We use the formalism of maximum likelihood estimation: we combine the structural and the node information into a global likelihood function and provide a highly scalable Expectation-Maximization algorithm for the estimation of parameters.

### Model description and notation

Consider a multilayer network of *N* nodes and *L* layers. This is a set of graphs $G = \{G^{(\alpha )}\left( {\mathscr {V}}, {\mathscr {E}}^{(\alpha )}\right) \}_{1\le \alpha \le L}$ defined on a set ${\mathscr {V}}$ of *N* vertices shared across $L\ge 1$ layers, and ${\mathscr {E}}^{(\alpha )}$ is the set of edges in the layer $\alpha$. Each layer $\alpha \in \{1,\dots , L\}$ is a graph $G^{(\alpha )}({\mathscr {V}}, {\mathscr {E}}^{(\alpha )})$ with adjacency matrix $A^{(\alpha )} = [a_{ij}^{(\alpha )}] \in {\mathbb {R}}^{N\times N}$, where $a_{ij}^{(\alpha )}$ is the number of edges of type $\alpha$ from *i* to *j*; here we consider only positive discrete entries; for binary entries, $E=\sum _{i,j,\alpha } a_{ij}^{(\alpha )}$ is the total the number of edges. Alternatively, we can consider a 3-way tensor *A* with dimensions $N\times N \times L$. In addition, for each node $i\in {\mathscr {V}}$ consider the vector of covariates $X_i \in {\mathbb {R}}^{1\times K}$ (alternatively called also attributes or metadata), where *K* is the total number of attributes. Here, for simplicity we focus on the case of $K=1$ and categorical covariates with *Z* different categories. However, we can easily generalize to more than one covariate by encoding each possible combination of them as a different value of one single covariate. For example, for two covariates being gender and nationality, we can encode $X_{i}$ being one covariate with possible values female/American, male/Spanish and so forth. One could also consider real-valued covariates by cutting them into bins. Nevertheless, a future expansion should include the possibility to work with any type of metadata.

A community is a subset of vertices that share some properties. Formally, each node belongs to a community to an extent measured by a *C*-dimensional vector denoted *membership*. Since we are interested in directed networks, for each node *i* we assign two such vectors, $u_i$ and $v_i$ (for undirected networks we set $u =v$); these determine how *i* forms outgoing and incoming links respectively. Each layer $\alpha$ has an *affinity* matrix $W^{(\alpha )} = [w_{kl}^{(\alpha )}]\in {\mathbb {R}}^{C \times C}$ which describes the density of edges between each pair (*k*, *l*) of groups. Each community $k\in \{1,\dots , C\}$ is linked to a category $z\in \{1,\dots , Z\}$ by a parameter $\beta _{kz}$, that explains how much information of the *z*-th category is used to create the *k*-th community. To summarize, we consider two types of observed data: the adjacency tensor $A = \{A^{(\alpha )}\}_{1\le \alpha \le L}$ and the design matrix $X=\{X_i\}_{i\in \{1,\dots , N\}}$; the first contains information about the networks topology structure, the latter about the node covariates. In addition, we have the model parameters that we compactly denote as $\varTheta =\left\{ U,V,W,\beta \right\}$.

**Figure 7:**
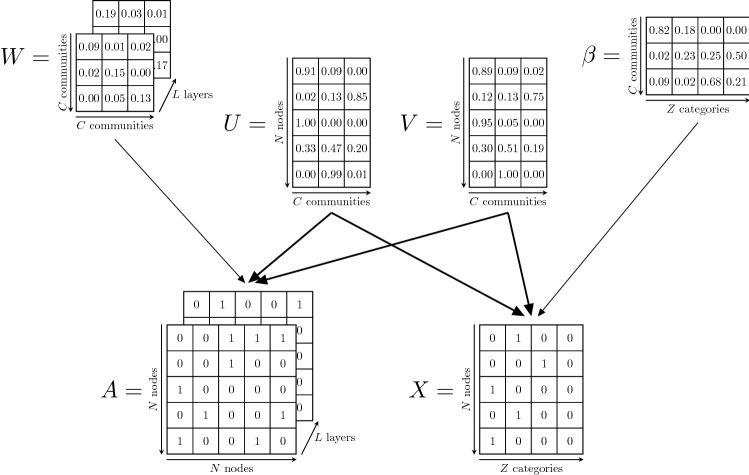
Graphical model representation of the algorithm MTCOV. *A* is the adjacency tensor, *X* is the design matrix and $W, U, V, \beta$ are the latent parameters $\varTheta$. The membership matrices *U* and *V* couple the two datasets, and this is highlighted by the stronger thickness; whereas *W* and $\beta$ are specific to the adjacency tensor and design matrix respectively. Here we present an example with binary adjacency matrix *A*, but the model is valid for more general weighted networks.

The goal is to find the latent parameters $$\varTheta$$ using the data *A* and *X*. In other words, given an observed multilayer network with adjacency tensor *A* and design matrix *X*, our goal is to simultaneously infer the node’s membership vectors $$u_i$$ and $$v_i$$$$\forall i\in \{1,\ldots ,N \}$$; the affinity matrices $$W^{(\alpha )}$$, $$\forall \alpha \in \{1,\dots , L\}$$, and the matrix $$\beta = [\beta _{kz}] \in {\mathbb {R}}^{C \times Z}$$, which captures correlations between communities and attributes. A visual overview of the proposed model is shown in Fig. 7. We consider a probabilistic generative model where MTCOV generates the network and the attributes probabilistically, assuming an underlying structure consisting of *C* overlapping communities. We adopt a maximum likelihood approach where, given the latent parameters $$\varTheta$$, we assume that the data *A* and *X* have independent likelihoods; in other words, we assume that *A* and *X* are *conditionally independent* given the latent parameters $$\varTheta$$. In addition, we assume that the memberships *U* and *V* couple the two datasets, as they are parameters shared between the two likelihoods; whereas the *W* and $$\beta$$ are specific to the adjacency and design matrix respectively. We describe separately the procedures for modeling the topology of the network and the node attributes and then we show how to combine them in a unified log-likelihood framework.

### Modeling the network topology

In modeling the likelihood of the network topology, we adopt the ideas behind MULTITENSOR: we assume that the expected number of edges of type $\alpha$ from *i* to *j* is given by the parameter:

1 $$\begin{aligned} M_{ij}^{(\alpha )}= \sum _{k,l =1}^C u_{ik}v_{jl}w_{kl}^{(\alpha )} . \end{aligned}$$

We then assume that each entry $a_{ij}^{(\alpha )}$ of the adjacency tensor is extracted from a Poisson distribution with parameter $M_{ij}^{(\alpha )}$. This is a common choice for network data^49–51^ as it leads to tractable and efficient algorithmic implementations, compared for instance with other approaches that use Bernoulli random variables^9,27^; it also allows the flexibility of treating both binary and integer-weighted networks. We further assume that, given the memberships and affinity matrices, the edges are distributed independently; this is again a conditional independence assumption.

We can then write the likelihood of the network topology as:

2 $$\begin{aligned} P_{G}(A|U,V, W) = \prod _{i,j =1}^N \prod _{\alpha =1}^L \frac{{e}^{-M_{ij}^{(\alpha )}} \left( M_{ij}^{(\alpha )}\right) ^{A_{ij}^{(\alpha )}}}{A_{ij}^{(\alpha )} !} , \end{aligned}$$

which leads to the log-likelihood ${\mathscr {L}}_G(U,V,W)$ for the *structural dimension*:

3 $$\begin{aligned} {\mathscr {L}}_G(U,V,W) = \sum _{i,j,\alpha } \bigg [ A_{ij}^{(\alpha )} \text {log} \sum _{k,l} u_{ik}v_{jl}w_{kl}^{(\alpha )} - \sum _{k,l} u_{ik}v_{jl}w_{kl}^{(\alpha )} \bigg ], \end{aligned}$$

where we have neglected constants that do not depend on the parameters.

### Modeling the node attributes

In modeling the likelihood of the attributes, we assume that this extra information is generated from the membership vectors; this captures the intuition that knowing a node’s community membership helps in predicting the value of the node’s attribute. This assumption has also been made in other models for single-layer attributed networks^9^ where one wants to enforce the tendency that nodes in the same community (for assortative structures) are likely to share common attributes. Different approaches^37,38^ assume instead independence between attributes and membership, which follows a different idea of observing an interaction between individuals if either they belong to the same community (for assortative structures) or they share an attribute or both.

Then, we model the probability of observing the *z*-th category for the attribute covariate of node *i* as the parameter:

4 $$\begin{aligned} \pi _{iz} = \frac{1}{2}\sum _{k=1}^C \beta _{kz} (u_{ik}+v_{ik}) , \end{aligned}$$

where $\beta _{kz}$ is the probability of observing a particular category *z* together with a community *k*; thus $\pi _i= (\pi _{i1} \dots , \pi _{iZ})$ is a *Z*-dimensional vector such that $\pi _{iz} \in \left[ 0,1\right]$ and $\sum _{z=1}^Z \pi _{iz} =1, \forall i$. For convenience, we consider one-hot encoding for $x_i = (x_{i1}, \dots , x_{iZ})$, the realization of the random variable $X_i$: $x_{iz}=1$ if node *i* has attribute corresponding to category *z*, 0 otherwise and $\sum _{z=1}^Z x_{iz} =1$; the original design matrix $X_{N\times 1}$ is thus translated into a binary matrix $X_{N\times Z}$.

We then assume that each entry $X_{i}$ of the design matrix is extracted from a multinomial distribution of parameter $\pi _{i}$, which yields the likelihood of the covariates:

5 $$\begin{aligned} P_{X}(X_i=x_i|U,V,\beta ) = P_{X}(X_{i1}=x_{i1}, \dots , X_{iZ}= x_{iZ}|U,V,\beta ) = \pi _{i1}^{x_{i1}} \dots \pi _{iZ}^{x_{iZ}}\ \ . \end{aligned}$$

In order to satisfy the sum constraint $\sum _{z=1}^Z \pi _{iz} =1$, we impose the normalizations $\sum _{z=1}^Z \beta _{kz} =1$, valid $\forall k$ and $\sum _{k=1}^C u_{ik}= \sum _{k=1}^C v_{ik} =1$, valid $\forall i$. Such constraints are a particular case for which the general constraint for the multinomial parameter is satisfied. Although they are not the only choices, they allow us to give a probabilistic meaning to the components of $\beta$ and the memberships *U* and *V*. As done for the network’s edges, we assume conditional independence for the attributes on the various nodes. This leads to the log-likelihood ${\mathscr {L}}_{X}(U,V,\beta )$ for the *attribute dimension*:

6 $$\begin{aligned} {\mathscr {L}}_{X}(U,V,\beta ) = \sum _{i=1}^N \sum _{z=1}^Z x_{iz} \, \text {log}(\pi _{iz}) =\sum _{i, z} x_{iz} \, \text {log}\bigg (\frac{1}{2}\sum _{k}\beta _{kz} (u_{ik}+v_{ik})\bigg ) . \end{aligned}$$

Note, we assume that the attributes have values that can be binned in a finite number *Z* of unordered categories and the attributes do not need to be one-dimensional. Indeed, we can encode each combination of more attributes as a different value of one-dimensional “super-attribute”. The model will not be affected, but the computational complexity might increase.

### Inference with the EM algorithm

Having described how the model works and its main assumptions and intuitions, we now turn our attention to describe how to fit the parameters to the data, in other words, how to perform inference. We assume conditional independence between the network and attribute variables, thus we can decompose the total log-likelihood into a sum of two terms ${\mathscr {L}}(U,V,W,\beta )={\mathscr {L}}_G(U,V,W)+ {\mathscr {L}}_{X}(U,V,\beta )$. However, in practice, we can improve parameters’ inference performance by better balancing the contributions of the two terms as their magnitude can be on different scales, thus the risk of biasing the total likelihood maximization towards one of the two terms. For this, we introduce a scaling parameter $\gamma \in [0,1]$ that explicitly controls the relative contribution of the two terms. The total log-likelihood is then:

7 $$\begin{aligned} {\mathscr {L}}(U,V,W,\beta )= & \, \left( 1- \gamma \right) {\mathscr {L}}_G(U,V,W)+ \gamma \, {\mathscr {L}}_{X}(U,V,\beta )\nonumber \\= & \, (1-\gamma ) \sum _{i,j,\alpha } \bigg [A_{ij}^{(\alpha )} \text {log} \sum _{k,l} u_{ik}v_{jl}w_{kl}^{(\alpha )} - \sum _{k,l} u_{ik}v_{jl}w_{kl}^{(\alpha )}\bigg ] \nonumber \\&+\gamma \, \sum _{i, z} x_{iz} \text {log}\bigg (\frac{1}{2}\sum _{k} \beta _{kz} (u_{ik}+v_{ik})\bigg ) . \end{aligned}$$

Varying $\gamma$ from 0 to 1 lets us interpolate between two extremes: analyzing the data purely in terms of the network topology or purely in terms of the attribute information. One can either fix this *a priori* based on the goal of the application, closer to 0 for link prediction or closer to 1 for attribute classification, or this can be treated as a hyperparameter that must be estimated, whose optimal value is obtained by fitting the data *via* tuning techniques (for instance cross-validation). This approach provides a natural quantitative measure for the dependence between the communities and the two sources of information. Notice that one can rescale *a priori* each likelihood term individually in order to control even more their magnitudes, and then add it to Eq. (7). This choice should be made based on the dataset at hand. Here we consider rescaling ${\mathscr {L}}_G$ and ${\mathscr {L}}_X$ only in studying the social support networks of Indian villages, as we have enough data for estimating the normalization coefficients; see Supplementary Section S3.1 for details.

We wish to find the $\varTheta = ({U,V, W,\beta })$ that maximizes Eq. (7). In general, this is computationally difficult, but we make it tractable by adopting a variational approach using an Expectation-Maximization (EM) algorithm^52^, similar to what done by De Bacco et al.^13^, but extended here to include attribute information. Namely, we introduce two probability distributions: $h_{ikz}$ and $\rho _{ijkl}^{(\alpha )}$. For each *i*, *z* with $X_{iz}=1$, $h_{izk}$ represents our estimate of the probability that the *i*-th node has the *z*-th category, given that it belongs to the community *k*. On the other hand, for each $i, j, \alpha$ with $A_{ij}^{(\alpha )} =1$, $\rho _{ijkl}^{(\alpha )}$ is the probability distribution over pairs of groups *k*, *l*.

Using Jensen’s inequality $\log {\bar{x}} \ge \overline{\log x}$ for each log-likelihood term gives:

8 $$\begin{aligned} {\mathscr {L}}_X(U,V,\beta )&\ge \sum _{i, z} x_{iz} \sum _{k} h_{izk}\log \frac{\beta _{kz} (u_{ik}+v_{ik})}{2h_{izk}} =\sum _{i,z,k} x_{iz} \left[ h_{izk}\,\log \beta _{kz} (u_{ik}+v_{ik}) - h_{izk}\, \log 2h_{izk}\right] \nonumber \\&= {\mathscr {L}}_X(U,V,\beta , h) \end{aligned}$$

9 $$\begin{aligned} {\mathscr {L}}_G(U,V,W)&\ge \sum _{i,j,k,l,\alpha }\bigg [ A_{ij}^{(\alpha )}\bigg (\rho _{ijkl}^{(\alpha )} \, \log u_{ik}v_{jl}w_{kl}^{(\alpha )} - \rho _{ijkl}^{(\alpha )}\log \rho _{ijkl}^{(\alpha )}\bigg ) - u_{ik}v_{jl}w_{kl}^{(\alpha )}\bigg ] = {\mathscr {L}}_G(U,V,W, \rho ). \end{aligned}$$

These lower bounds hold with equality when

10 $$\begin{aligned} h_{izk}=\frac{\beta _{kz} (u_{ik}+v_{ik})}{\sum _{k'} \beta _{k'z} (u_{ik'}+v_{ik'})},\quad \rho _{ijkl}^{(\alpha )} =\frac{u_{ik}v_{jl}w_{kl}^{(\alpha )}}{\sum _{k',l'} u_{ik'}v_{jl'}w_{k'l'}^{(\alpha )}}, \end{aligned}$$

thus maximizing ${\mathscr {L}}_X(U,V,\beta )$ is equivalent to maximizing ${\mathscr {L}}_X(U,V,\beta , h)$; similarly for ${\mathscr {L}}_G(U,V,W)$ and ${\mathscr {L}}_G(U,V,W, \rho )$ (this was also the same result derived by De Bacco et al.^13^). Overall, we aim at maximizing ${\mathscr {L}}(U,V,W,\beta , h,\rho )=\left( 1- \gamma \right) {\mathscr {L}}_G(U,V,W, \rho )+ \gamma \, {\mathscr {L}}_{X}(U,V,\beta ,h)$, in analogy with what was done before. These maximizations can be performed by alternatively updating a set of parameters while keeping the others fixed. The EM algorithm performs these steps by alternatively updating *h*, $\rho$ (Expectation step) and $\varTheta$ (Maximization step); this is done starting from a random configuration until ${\mathscr {L}}(\varTheta , h,\rho )$ reaches a fixed point. Calculating Eq. (10) represents the E-step of the algorithm. The M-step is obtained by computing partial derivatives of ${\mathscr {L}}(\varTheta , h,\rho )$ with respect to the various parameters in $\varTheta$ and setting them equal to zero. We add Lagrange multipliers $\lambda =\left( \lambda ^{(\beta )},\lambda ^{(u)}, \lambda ^{(v)}\right)$ to enforce constraints:

11 $$\begin{aligned} {\mathscr {L}}^{'}(\varTheta , h,\rho ,\lambda )= {\mathscr {L}}(\varTheta , h,\rho ) - \sum _{k} \lambda ^{(\beta )}_{k} \left( \sum _{z=1}^Z \beta _{kz} -1\right) - \sum _{i} \lambda ^{(u)}_{i} \left( \sum _{k=1}^C u_{ik} -1\right) - \sum _{i} \lambda ^{(v)}_{i} \left( \sum _{k=1}^C v_{ik} -1\right) . \end{aligned}$$

For instance, focusing on the update for $\beta _{zk}$, setting the derivative with respect to it in Eq. (11) to zero and enforcing the constraint $\sum _{z=1}^Z \beta _{kz} =1$ gives $\lambda ^{(\beta )}_k = \gamma \sum _{i,z} x_{iz} h_{izk}$; plugging this back finally gives:

12 $$\begin{aligned} \beta _{kz} = \frac{\sum _{i} \,x_{iz} \,h_{izk}}{\sum _{i,z} \,x_{iz} \,h_{izk}} , \end{aligned}$$

which is valid for $\gamma \ne 0$. Doing the same for the other parameters yields (see Supplementary Section S1 for details):

13 $$\begin{aligned} u_{ik}= & \, \frac{\gamma \, \sum _z \,x_{iz} h_{izk} + (1-\gamma )\, \sum _{j,l,\alpha }A_{ij}^{(\alpha )} \rho _{ijkl}^{(\alpha )}}{ \gamma + (1-\gamma ) \sum _{j,\alpha }A_{ij}^{(\alpha )}} \end{aligned}$$

14 $$\begin{aligned} v_{ik}= & \, \frac{\gamma \,\sum _z\, x_{iz} h_{izk} + (1-\gamma )\, \sum _{j,l,\alpha }A_{ji}^{(\alpha )} \rho _{jilk}^{(\alpha )}}{ \gamma + (1-\gamma ) \sum _{j,\alpha }A_{ji}^{(\alpha )}} \end{aligned}$$

15 $$\begin{aligned} w_{kl}^{(\alpha )}= & \, \frac{\sum _{i,j} A_{ij}^{(\alpha )} \rho _{ijkl}^{(\alpha )}}{\sum _i u_{ik} \sum _j v_{jl}} \quad , \end{aligned}$$

where Eq. (15) is valid for $\gamma \ne 1$. The EM algorithm thus consists in randomly initializing the parameters $\varTheta$ and then repeatedly alternating between updating *h* and $\rho$ using Eq. (10) and updating $\varTheta$ using Eqs. (12)–(15) until ${\mathscr {L}}(\varTheta , h,\rho )$ reaches a fixed point. A pseudo-code is given in Algorithm 1. In general, the fixed point is a local maximum but we have no guarantees that it is also the global one. In practice, we run the algorithm several times, starting from different random initializations and taking the run with the largest final ${\mathscr {L}}(\varTheta , h,\rho )$. The computational complexity per iteration scales as $O(M\,C^{2}+NCZ)$, where *M* is the total number of edges summed across layers. In practice, *C* and *Z* have similar order of magnitude, usually much smaller than the system size *M*; for sparse networks, as is often the case for real datasets, $M \propto N$, thus the algorithm is highly scalable with a total running time linear in the system size. An experimental analysis of the computational time is provided in the Supplementary Section S2.

Notice that, although we started from a network log-likelihood ${\mathscr {L}}_{G}(U,V,W)$ similar to the one proposed in the MULTITENSOR model^13^, the only update preserved from that is the one of $w_{kl}$ in Eq. (15). The updates for $u_{ik}$ and $v_{ik}$ are instead quite different; the main reason is that here we incorporated the node attributes, which appear both explicitly and implicitly (through *h*) inside the updates. In addition, here we enforce normalizations like $\sum _{k}u_{ik}=1$, not enforced in MULTITENSOR. This implies that our model restricted to $\gamma =0$, i.e., no attribute information, does not correspond exactly to MULTITENSOR. This also implies that, upon convergence, we can directly interpret the memberships as *soft* community assignments (or overlapping) without the need of post-processing their values; in words, $u_{ik}$ represent the probability of node *i* to belong to the *outgoing* community *k*, similarly for $v_{ik}$ and an *incoming* membership. This distinction is necessary when considering directed networks. If one is interested in recovering *hard* memberships, where a node is assigned to only one community, then one can choose the community corresponding to the maximum entry of *u* or *v*.

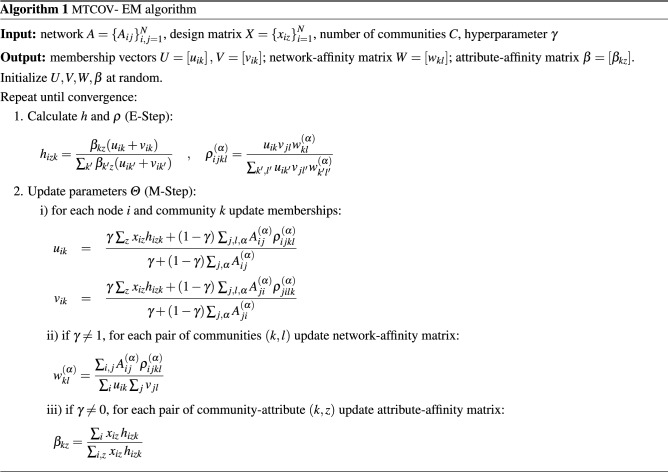



### Evaluation metrics

We adopt two different criteria for performance evaluation, based on having or not having access to ground-truth values for the community assignments. The first case applies to synthetic-generated data, the second to both synthetic and real-world data. We explain performance metrics in detail below.

#### Ground-truth available

In the presence of a known partition, we measure the agreement between the set of ground-truth communities ${\mathscr {C}}^*$ and the set of detected communities ${\mathscr {C}}$ using metrics for recovering both hard and soft assignments. For hard partitions, the idea is to match every detected community with its most similar ground-truth community and measure similarity $\delta ({\mathscr {C}}_i^*, {\mathscr {C}}_j)$ (and vice versa for every ground-truth community matched against a detected community) as done by Yang et al.^9^. The final performance is the average of these two comparisons:

16 $$\begin{aligned} \frac{1}{2|{\mathscr {C}}^*|} \sum _{{\mathscr {C}}_i^*\in {\mathscr {C}}^*} \max _{{\mathscr {C}}_j\in {\mathscr {C}}} \delta ({\mathscr {C}}_i^*, {\mathscr {C}}_j) + \frac{1}{2|{\mathscr {C}}|} \sum _{{\mathscr {C}}_j\in {\mathscr {C}}} \max _{{\mathscr {C}}_i^*\in {\mathscr {C}}^*} \delta ({\mathscr {C}}_i^*, {\mathscr {C}}_j) , \end{aligned}$$

where here we consider as similarity metric $\delta (\cdot )$ the F1-score and the Jaccard similarity.

In both cases, the final score is a value between 0 and 1, where 1 indicates the perfect matching between detected and ground-truth communities. For soft partitions, we consider two standard metrics for measuring distance between vectors as done by De Bacco et al.^13^, such as *cosine similarity* (CS) and $L_{1}$ error, averaged over the nodes:

17 $$\begin{aligned} CS(U,U^{0})= & \, \frac{1}{N} \sum _{i=1}^{N} \frac{u_{i} \cdot u^{0}_{i}}{||u_{i}||_{2}\,||u^{0}_{i}||_{2}} =\frac{1}{N} \sum _{i=1}^{N} \sum _{k=1}^{C} \frac{u_{ik} \, u^{0}_{ik}}{||u_{i}||_{2}\,||u^{0}_{i}||_{2}} \end{aligned}$$

18 $$\begin{aligned} L_{1}(U,U^{0})= & \, \frac{1}{2N} \sum _{i=1}^{N}|| u_{i}-u_{i}^{0}||_{1}=\frac{1}{2N} \sum _{i=1}^{N}\sum _{k=1}^{C}| u_{ik}-u_{ik}^{0}| , \end{aligned}$$

where $u_{i}$ is the *C*-dimensional vector containing the *i*-th row of *U*, representing the detected membership and similarly for $u_{i}^{0}$ for the ground-truth $U^{0}$. The factor 1/2 ensures that the $L_{1}$ distance ranges from 0 for identical distributions to 1 for distributions with disjoint support. Similarly to the what done for hard partitions, we match the ground-truth and detected communities by choosing the permutation of *C* groups that gives the highest cosine similarity or smallest $L_{1}$ distance.

#### Ground-truth not available

In the absence of ground-truth, these metrics cannot be computed, and one must resort to other approaches for model evaluation. Here we consider performance in prediction tasks when hiding part of the input datasets while fitting the parameters, and in particular on the extent to which partial knowledge of network edges helps predict node attributes and vice versa. Thus we consider a measure for link-prediction and one for correct retrieval of the attributes. For link-prediction, we used the AUC statistic, equivalent to the area under the receiver-operating characteristic (ROC) curve^53^. It represents the probability that a randomly chosen missing connection (a true positive) is given a higher score than a randomly chosen pair of unconnected vertices (a true negative). Thus, an AUC statistic equal to 0.5 indicates random chance, while the closer it is to 1, the more our model’s predictions are better than chance. We measure the probability of observing an edge as the predicted expected Poisson parameters of Eq. (1). For the attribute, instead, we use the accuracy as a quality measure. For each node, we compute the predicted expected multinomial parameter $\pi _i$ using Eq. (4). We then assign to each node the category with the highest probability, computing the accuracy as the ratio between the correctly classified examples over the total number of nodes. As baselines, we compare with the accuracy obtained with a random uniform probability and the highest relative frequency observed in the training set.

### Cross-validation tests and hyperparameter settings

We perform prediction tasks using cross-validation with 80–20 splits: we use 80% of the data for training the parameters and then measure AUC and accuracy on the remaining 20% test set. Specifically, for the network topology, we hold out 20% of the triples $(i,j,\alpha )$; for the attributes, we hold out 20% of the entries of the categorical vector.

Our model has two hyperparameters, the scaling parameter $\gamma$ and the number of communities *C*. We estimate them by using 5-fold cross-validation along with grid search to range across their possible values. We then select the combination (${\hat{C}},{\hat{\gamma }}$) that returns the best average performance over the cross-validation runs. Standard cross-validation considers performance in terms of a particular metric. However, here we have two possible ones which are qualitatively different, i.e., AUC and accuracy. Depending on the task at hand, one can define performance as a combination of the two, bearing in mind that the values of (${\hat{C}},{\hat{\gamma }})$ at the maximum of either of them might not coincide. Here we select (${\hat{C}},{\hat{\gamma }})$ as the values are jointly closer to both the maximum values. In the experiments where one of the two hyperparameters is fixed *a priori*, we run the same procedure but vary with grid search only the unknown hyperparameter.

## Supplementary information

Supplementary Information.

## Data Availability

The code used for the analysis and to generate the synthetic data is publicly available and can be found at https://github.com/mcontisc/MTCOV.

## Code availabilty

An open-source algorithmic implementation available at https://github.com/mcontisc/MTCOV.

## References

[1] Waskiewicz, T. Friend of a friend influence in terrorist social networks. In *Proceedings on the international conference on artificial intelligence (ICAI)*, 1 (The Steering Committee of The World Congress in Computer Science, Computer..., 2012).

[2] Pinheiro, C. A. R. Community detection to identify fraud events in telecommunications networks. In *SAS SUGI proceedings: customer intelligence* (2012).

[3] Pan W-F, Jiang B, Li B (2013). Refactoring software packages via community detection in complex software networks. Int. J. Autom. Comput..

[4] Bechtel JJ (2005). Lung cancer detection in patients with airflow obstruction identified in a primary care outpatient practice. Chest.

[5] Chen J, Zhang H, Guan Z-H, Li T (2012). Epidemic spreading on networks with overlapping community structure. Physica A Stat. Mech. Appl..

[6] Traud AL, Kelsic ED, Mucha PJ, Porter MA (2011). Comparing community structure to characteristics in online collegiate social networks. SIAM Rev..

[7] Newman ME (2006). Modularity and community structure in networks. Proc. Natl. Acad. Sci..

[8] Peel L, Larremore DB, Clauset A (2017). The ground truth about metadata and community detection in networks. Sci. Adv..

[9] Yang, J., McAuley, J. & Leskovec, J. Community detection in networks with node attributes. In *2013 IEEE 13th international conference on data mining*, 1151–1156 (IEEE, 2013).

[10] Falih I, Grozavu N, Kanawati R, Bennani Y (2018). Community detection in attributed network. Companion Proc. Web Conf..

[11] Fortunato S (2010). Community detection in graphs. Phys. Rep..

[12] De Domenico M (2013). Mathematical formulation of multilayer networks. Phys. Rev. X.

[13] De Bacco C, Power EA, Larremore DB, Moore C (2017). Community detection, link prediction, and layer interdependence in multilayer networks. Phys. Rev. E.

[14] Schein, A., Paisley, J., Blei, D. M. & Wallach, H. Bayesian Poisson tensor factorization for inferring multilateral relations from sparse dyadic event counts. In *Proceedings of the 21st ACM SIGKDD international conference on knowledge discovery and data mining*, 1045–1054 (2015).

[15] Schein, A., Zhou, M., Blei, D. M. & Wallach, H. Bayesian Poisson tucker decomposition for learning the structure of international relations. In *Proceedings of the 33rd international conference on machine learning*, vol. 48 (2016).

[16] Valles-Catala T, Massucci FA, Guimera R, Sales-Pardo M (2016). Multilayer stochastic block models reveal the multilayer structure of complex networks. Phys. Rev. X.

[17] Stanley N, Shai S, Taylor D, Mucha P (2016). Clustering network layers with the strata multilayer stochastic block model. IEEE Trans. Netw. Sci. Eng..

[18] Peixoto TP (2015). Inferring the mesoscale structure of layered, edge-valued, and time-varying networks. Phys. Rev. E.

[19] Paul S, Chen Y (2016). Consistent community detection in multi-relational data through restricted multi-layer stochastic blockmodel. Electron. J. Stat..

[20] Gheche ME, Chierchia G, Frossard P (2020). Orthonet: multilayer network data clustering. IEEE Trans. Signal Inf. Process. Netw..

[21] Papadopoulos, A., Rafailidis, D., Pallis, G. & Dikaiakos, M. D. Clustering attributed multi-graphs with information ranking. In *Proceedings, Part I, of the 26th international conference on database and expert systems applications—volume 9261, DEXA 2015*, 432–446 (Springer, 2015).

[22] Papadopoulos A, Pallis G, Dikaiakos MD (2017). Weighted clustering of attributed multi-graphs. Computing.

[23] Chang, S. *et al.* Heterogeneous network embedding via deep architectures. In *Proceedings of the 21th ACM SIGKDD international conference on knowledge discovery and data mining, KDD ’15*, 119–128 (2015).

[24] Sachan, M., Contractor, D., Faruquie, T. A. & Subramaniam, L. V. Using content and interactions for discovering communities in social networks. In *Proceedings of the 21st international conference on world wide web, WWW ’12*, 331–340 (2012).

[25] Sweet TM, Zheng Q (2018). Estimating the effects of network covariates on subgroup insularity with a hierarchical mixed membership stochastic blockmodel. Soc. Netw..

[26] Signorelli M, Wit EC (2019). Model-based clustering for populations of networks. Stat. Model..

[27] Newman ME, Clauset A (2016). Structure and inference in annotated networks. Nat. Commun..

[28] Bothorel C, Cruz JD, Magnani M, Micenkova B (2015). Clustering attributed graphs: models, measures and methods. Netw. Sci..

[29] Zhang Y, Levina E, Zhu J (2016). Community detection in networks with node features. Electron. J. Stat..

[30] Hric D, Peixoto TP, Fortunato S (2016). Network structure, metadata, and the prediction of missing nodes and annotations. Phys. Rev. X.

[31] Stanley N, Bonacci T, Kwitt R, Niethammer M, Mucha PJ (2019). Stochastic block models with multiple continuous attributes. Appl. Netw. Sci..

[32] Emmons S, Mucha PJ (2019). Map equation with metadata: varying the role of attributes in community detection. Phys. Rev. E.

[33] Xu, Z., Ke, Y., Wang, Y., Cheng, H. & Cheng, J. A model-based approach to attributed graph clustering. In *Proceedings of the 2012 ACM SIGMOD international conference on management of data*, 505–516 (2012).

[34] Bu Z, Li H-J, Cao J, Wang Z, Gao G (2017). Dynamic cluster formation game for attributed graph clustering. IEEE Trans. Cybern..

[35] Tallberg C (2004). A bayesian approach to modeling stochastic blockstructures with covariates. J. Math. Sociol..

[36] White A, Murphy TB (2016). Mixed-membership of experts stochastic blockmodel. Netw. Sci..

[37] Airoldi EM, Choi DS, Wolfe PJ (2011). Confidence sets for network structure. Stat. Anal. Data Min. ASA Data Sci. J..

[38] Sweet TM (2015). Incorporating covariates into stochastic blockmodels. J. Educ. Behav. Stat..

[39] Taylor D, Shai S, Stanley N, Mucha PJ (2016). Enhanced detectability of community structure in multilayer networks through layer aggregation. Phys. Rev. Lett..

[40] Taylor D, Caceres RS, Mucha PJ (2017). Super-resolution community detection for layer-aggregated multilayer networks. Phys. Rev. X.

[41] Holland PW, Laskey KB, Leinhardt S (1983). Stochastic blockmodels: first steps. Soc. Netw..

[42] Power, E. A. *Building Bigness: Religious Practice and Social Support in Rural South India*. Doctoral Dissertation, Stanford University, Stanford, CA (2015).

[43] Power EA (2017). Social support networks and religiosity in rural South India. Nat. Hum. Behav..

[44] Power EA, Ready E (2019). Cooperation beyond consanguinity: post-marital residence, delineations of kin and social support among South Indian Tamils. Philos. Trans. R. Soc. B Biol. Sci..

[45] McAuley, J. & Leskovec, J. Learning to discover social circles in ego networks. In *Proceedings of the 25th international conference on neural information processing systems—volume 1, NIPS’12*, 539–547 (2012).

[46] Girvan M, Newman MEJ (2002). Community structure in social and biological networks. Proc. Natl. Acad. Sci..

[47] Adamic, L. A. & Glance, N. The political blogosphere and the 2004 U.S. election: divided they blog. In *Proceedings of the 3rd international workshop on link discovery, LinkKDD ’05*, 36–43 (2005).

[48] Kolda TG, Bader BW (2009). Tensor decompositions and applications. SIAM Rev..

[49] Ball B, Karrer B, Newman MEJ (2011). Efficient and principled method for detecting communities in networks. Phys. Rev. E.

[50] Gopalan PK, Blei DM (2013). Efficient discovery of overlapping communities in massive networks. Proc. Natl. Acad. Sci. USA.

[51] Gopalan, P., Hofman, J. M. & Blei, D. M. Scalable recommendation with hierarchical poisson factorization. In *Proceedings of the 31-st conference on uncertainty in artificial intelligence*, 122–129 (2015).

[52] Dempster AP, Laird NM, Rubin DB (1977). Maximum likelihood from incomplete data via the em algorithm. J. R. Stat. Soc. Ser. B (Methodol.).

[53] Hanley JA, McNeil BJ (1982). The meaning and use of the area under a receiver operating characteristic (roc) curve. Radiology.

