# Supplementary material for: Community detection with node attributes in multilayer networks
Source: Sci Rep. 2020 Sep 25;10:15736. doi: 10.1038/s41598-020-72626-y (PMC7519123; doi:10.1038/s41598-020-72626-y)
Supplement: Supplementary file 1 — Supplementary Information. [file 41598_2020_72626_MOESM1_ESM.pdf]

Supplement: “Community detection with node attributes in multilayer networks”.

Martina Contisciani[1,*], Eleanor Power[2,†], and Caterina De Bacco[1,+]

[1]Max Planck Institute for Intelligent Systems, Cyber Valley, 72076, Tübingen, Germany
[2]London School of Economics and Political Science, Department of Methodology, London, WC2A 2AE, United Kingdom
[*]martina.contisciani@tuebingen.mpg.de
[†]e.a.power@lse.ac.uk
[+]caterina.debacco@tuebingen.mpg.de

S1 Methods: EM detailed derivation

We show derivations of the updates given in equations (12)-(15) of the main manuscript.

The partial derivative with respect to the elements of the affinity matrices $W^{(\alpha)}$ is given by

$$\frac{\partial \mathscr{L}}{\partial w_{kl}^{(\alpha)}} = (1-\gamma)\frac{\partial \mathscr{L}_G}{\partial w_{kl}^{(\alpha)}} = (1-\gamma)\sum_{i,j}\left[\frac{A_{ij}^{(\alpha)}\rho_{ijkl}^{(\alpha)}}{w_{kl}^{(\alpha)}} - u_{ik}v_{jl}\right] \quad . \tag{S1}$$

The valid update when γ is different from 1, is given by setting equation (S1) to zero and we obtain

$$w_{kl}^{(\alpha)} = \frac{\sum_{i,j} A_{ij}^{(\alpha)}\rho_{ijkl}^{(\alpha)}}{\sum_i u_{ik}\sum_j v_{jl}} \quad . \tag{S2}$$

In order to take the derivative with respect to β_{kz} we need to consider the Lagrange multiplier $\lambda_k^{(\beta)}$ because of the constraint in equation (11). Then,

$$\frac{\partial \mathscr{L}}{\partial \beta_{kz}} = \gamma\left(\frac{1}{\beta_{kz}}\sum_i x_{iz}h_{izk}\right) - \lambda_k^{(\beta)} \quad , \tag{S3}$$

and setting it to zero implies

$$\beta_{kz} = \frac{\gamma}{\lambda_k^{(\beta)}}\sum_i x_{iz}h_{izk} \quad . \tag{S4}$$

Enforcing the constraint (11), we have

$$\sum_z \frac{\gamma}{\lambda_k^{(\beta)}}\sum_i x_{iz}h_{izk} = 1 \quad , \tag{S5}$$

which implies

$$\lambda_k^{(\beta)} = \gamma\sum_{i,z} x_{iz}h_{izk} \quad . \tag{S6}$$

Plugging (S6) into (S4), we obtain the update:

$$\beta_{kz} = \frac{\sum_i x_{iz}h_{izk}}{\sum_{i,z} x_{iz}h_{izk}} \quad . \tag{S7}$$

Focusing the attention on the elements of the matrix U, we first consider that plugging the update for $w_{kl}^{(\alpha)}$ given in equation (S2) into the log-likelihood of the structural dimension $\mathscr{L}_G$, the last term becomes a constant. Indeed,

$$
\begin{aligned}
-\sum_{i,j}\sum_{k,l} u_{ik} v_{jl} \frac{\sum_{i,j} A_{ij}^{(\alpha)} \rho_{ijkl}^{(\alpha)}}{\sum_i u_{ik} \sum_j v_{jl}} &= -\sum_{k,l} \left(\frac{\sum_{i,j} A_{ij}^{(\alpha)} \rho_{ijkl}^{(\alpha)}}{\sum_i u_{ik} \sum_j v_{jl}} \sum_{i,j} u_{ik} v_{jl} \right) \\
&= -\sum_{k,l} \left(\sum_{i,j} A_{ij}^{(\alpha)} \rho_{ijkl}^{(\alpha)} \right) \\
&= -\sum_{i,j} A_{ij}^{(\alpha)} \sum_{k,l} \rho_{ijkl}^{(\alpha)} \\
&= -\sum_{i,j} A_{ij}^{(\alpha)} \\
&= -T^{(\alpha)} \qquad \text{(S8)}
\end{aligned}
$$

since $\sum_{k,l} \rho_{ijkl}^{(\alpha)} = 1$ and $\sum_{i,j} A_{ij}^{(\alpha)}$ is the number of links in layer α when the network is directed (or twice this value in the undirected case). Thus, we can ignore this term when estimating u_{ik}. Using the same strategy used in computing the update of β, we compute the Lagrange multiplier $\lambda_i^{(u)}$ for the constraint in equation (11). Then,

$$
\frac{\partial \mathscr{L}}{\partial u_{ik}} = \frac{1}{u_{ik}} \left(\gamma \sum_z x_{iz} h_{izk} + (1-\gamma) \sum_{j,l,\alpha} A_{ij}^{(\alpha)} \rho_{ijkl}^{(\alpha)} \right) - \lambda_i^{(u)} \qquad \text{(S9)}
$$

and

$$
u_{ik} = \frac{1}{\lambda_i^{(u)}} \left(\gamma \sum_z x_{iz} h_{izk} + (1-\gamma) \sum_{j,l,\alpha} A_{ij}^{(\alpha)} \rho_{ijkl}^{(\alpha)} \right) \quad . \qquad \text{(S10)}
$$

Enforcing the constraint $\sum_k u_{ik} = 1$ yields:

$$
\sum_k \frac{1}{\lambda_i^{(u)}} \left(\gamma \sum_z x_{iz} h_{izk} + (1-\gamma) \sum_{j,l,\alpha} A_{ij}^{(\alpha)} \rho_{ijkl}^{(\alpha)} \right) = 1 \qquad \text{(S11)}
$$

which implies

$$
\begin{aligned}
\lambda_i^{(u)} &= \sum_k \left(\gamma \sum_z x_{iz} h_{izk} + (1-\gamma) \sum_{j,l,\alpha} A_{ij}^{(\alpha)} \rho_{ijkl}^{(\alpha)} \right) \\
&= \gamma + (1-\gamma) \sum_{j,\alpha} A_{ij}^{(\alpha)} \quad , \qquad \text{(S12)}
\end{aligned}
$$

since $\sum_k h_{izk} = 1$, $\sum_{k,l} \rho_{ijkl}^{(\alpha)} = 1$ and $\sum_z x_{iz} = 1$. Plugging the result of equation (S12) into the equality (S10) we obtain

$$
u_{ik} = \frac{\gamma \sum_z x_{iz} h_{izk} + (1-\gamma) \sum_{j,l,\alpha} A_{ij}^{(\alpha)} \rho_{ijkl}^{(\alpha)}}{\gamma + (1-\gamma) \sum_{j,\alpha} A_{ij}^{(\alpha)}} \quad . \qquad \text{(S13)}
$$

In order to compute the update for V, we fix j and l, rewriting the attribute dimension $\mathscr{L}_X(U,V,\beta,h)$ as

$$
\mathscr{L}_X(U,V,\beta,h) = \sum_{j,z,l} x_{jz} (h_{jzl} \log(\beta_{lz}(u_{jl} + v_{jl})) - h_{jzl} \log(h_{jzl})) \quad . \qquad \text{(S14)}
$$

Analogously to before, we consider the Lagrange multiplier to satisfy the constraint given in equation (11), and we obtain

$$
v_{jl} = \frac{\gamma \sum_z x_{jz} h_{jzl} + (1-\gamma) \sum_{i,k,\alpha} A_{ij}^{(\alpha)} \rho_{ijkl}^{(\alpha)}}{\gamma + (1-\gamma) \sum_{i,\alpha} A_{ij}^{(\alpha)}} \quad . \qquad \text{(S15)}
$$

In each iteration of the EM algorithm, the parameters in $\Theta = (U,V,W,\beta)$ are updated with equations (S2), (S7), (S13) and (S15), until the log-likelihood $\mathscr{L}(\Theta,h,\rho)$ reaches a fixed point.

S2 Computational complexity

Based on Algorithm 1 in the main manuscript, the computational complexity per iteration of MTCOV scales as $O(MC^2 + NCZ)$, where M is the number of edges summed across layers, C the number of communities, N the number of nodes and Z the number of categories of the categorical attribute. In practice, C and Z have similar order of magnitude, usually much smaller than the system size M; for sparse networks, as is often the case for real datasets, $M \propto N$, thus the algorithm is highly scalable with a total running time linear in the system size. Figure S1 shows the empirical study of the computational complexity on large scale synthetic networks, both single and multilayer.
We generate synthetic multilayer networks as described in the Results section of the main manuscript, in the subsection Multilayer synthetic networks with ground truth. We generate directed networks with $L = 4$ layers, one assortative, one disassortative, one core-periphery and one with biased directed structure. The number of categories of the categorical attribute is $Z = 2$ and the match between attributes and planted communities is equal to 0.5. We vary the number of nodes $N \in \{100, 500, 1000, 5000, 10000, 50000\}$ and the number of communities $C \in \{2, 5, 10\}$. We run MTCOV with 5 different random initializations and the average results are shown in Fig. S1 (a). The black lines are the baseline for the quadratic (solid line) and the linear (dashed line) complexity in N, with $C = 5$ (similar results are obtained with different values of C). As expected, the total iteration time of MTCOV is linear in the system size.
For single-layer networks, we generate synthetic data by using the approach described in the subsection S4.1. We generate undirected networks with $C = 2$ communities, $Z = 2$ categories and a match between attributes and planted communities equal to 0.5. The $p_{in} = \frac{16}{N}$, $p_{out} = \frac{4}{N}$ and the number of nodes varies $N \in \{100, 500, 1000, 5000, 10000, 50000, 100000\}$. Figure S1 (b) shows the performance of MTCOV in comparison to CESNA and NC; the results are averaged over 5 random initializations. CESNA has convergence issues for networks with $N > 1000$, which results in unreliable community detection on large networks, hence we omit those results. NC and MTCOV show a linear computational complexity in the system size.

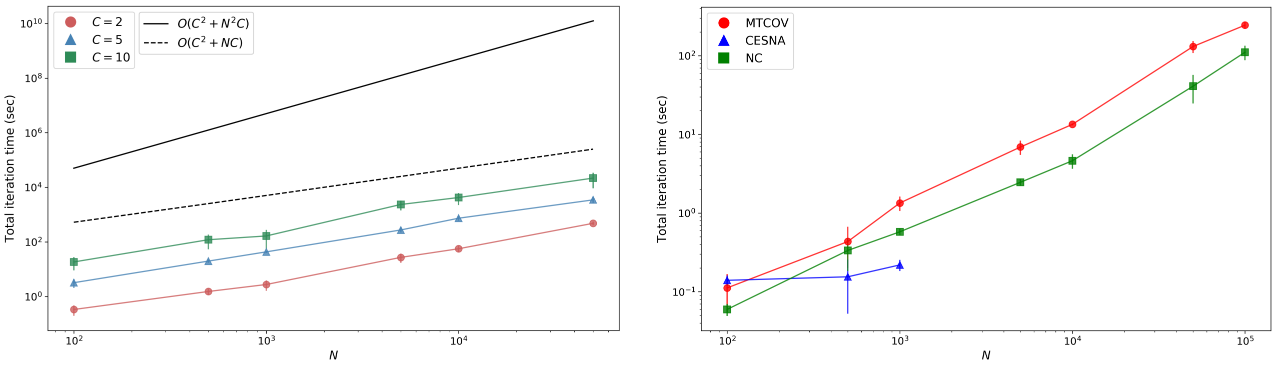

(a) Multilayer networks **(b)** Single-layer networks

Figure S1. Computational complexity. (a) Total iteration time of MTCOV on synthetic multilayer networks. (b) Total iteration time of MTCOV, CESNA and NC on synthetic single-layer networks. Results are averages and standard deviations over 5 different random initial conditions.

S3 Multilayer social support network of rural Indian villages

S3.1 Normalization

A way to control the magnitude of the likelihood terms is rescaling each term individually. Here, we estimate two linear regression models in order to obtain the normalization constants for the two terms. Given the access to several network datasets of the same kind (social support networks in Indian villages), we collect log-likelihood values with respect to the number of nodes (N), edges (E) of the observed network and the number of categories of the categorical attribute (Z). Quantitatively, this means normalizing as:

$$\mathscr{L} = (1-\gamma)\frac{\mathscr{L}_G}{c_N^G N + c_E^G E + c_Z^G Z} + \gamma \frac{\mathscr{L}_X}{c_N^X N + c_E^X E + c_Z^X Z} \ . \tag{S16}$$

The super and the subscripts of the c parameters indicate the dependent variable ($\mathscr{L}_X$ or $\mathscr{L}_G$) and the input regressor they refer to, respectively. In particular, we collect the data by running the model for each pair of network and categorical attribute,

arbitrarily fixing the number of communities $C = 3$ and the scaling parameter $\gamma = 0.5$. Table S1 states the values of the statistically significant coefficients for the estimation of the log-likelihood terms, and only those coefficients are used in the normalized equation (S16).

	$\mathscr{L}_X$	$\mathscr{L}_G$
c_N	-0.486^{***}	-1.778^{**}
c_E		-6.158^{***}
c_Z	-33.862^{***}	

Table S1. Coefficient estimates c_N^X, c_E^X, c_Z^X, c_N^G, c_E^G and c_Z^G for the two linear regression models.

On one side, this procedure allows to obtain coefficient estimates useful for analyzing all four networks of Indian villages in a quantitative and automatized way. On the other side, we are aware that these coefficients are strictly related to the type of network we are working with, i.e. their values cannot be used in network datasets other than the social support networks used here. Future works should investigate an automated normalization procedure applicable to any network dataset as a pre-processing step.

S3.2 Hyperparameter fitting

		Hyperparameters setting			
**Attribute**	**Method**	**Ala 2013**	**Ala 2017**	**Ten 2013**	**Ten 2017**
	MULTITENSOR	$C = 8$	$C = 9$	$C = 3$	$C = 3$
Caste	MTCOV	$C = 7, \gamma = 0.8$	$C = 7, \gamma = 0.8$	$C = 6, \gamma = 0.8$	$C = 6, \gamma = 0.9$
Religion	MTCOV	$C = 6, \gamma = 0.8$	$C = 6, \gamma = 0.8$	$C = 6, \gamma = 0.7$	$C = 6, \gamma = 0.7$
Age	MTCOV	$C = 8, \gamma = 0.4$	$C = 8, \gamma = 0.3$	$C = 9, \gamma = 0.2$	$C = 7, \gamma = 0.3$
Gender	MTCOV	$C = 10, \gamma = 0.8$	$C = 10, \gamma = 0.7$	$C = 10, \gamma = 0.4$	$C = 9, \gamma = 0.7$

Table S2. Values of the hyperparameters C and γ extracted by 5-fold cross-validation combined with grid-search.

S3.3 Biased link prediction

We use sampling bias techniques to assign higher or lower sampling probability to the entries of the adjacency tensor, which correspond to edges and non-edges. By defining *tpe* the total probability of selecting one edge (non-zero entry), we assign to the entries $a_{ij}^{(\alpha)} > 0$ the probability of being selected in the test set given by:

$$p_1 = \frac{tpe}{E} \quad , \tag{S17}$$

and for 0 entries

$$p_2 = \frac{1 - tpe}{N^2 L - E} \quad , \tag{S18}$$

where E and $N^2 L$ are the total number of the edges and entries of the adjacency tensor respectively. These two probabilities are assigned to the entries $a_{ij}^{(\alpha)}$ to perform a biased selection while choosing test and train sets, as in a selection of a binary mask. The *tpe* is used to select entries for the test set; in case of selecting entries uniformly at random, this value would be around 0.004. This low value is due to the common case in real networks of having sparse matrices, where the number of non-zero entries is much lower than the number of zeros. We create three different situations, starting from $tpe = 0.001$ where the probability of selecting one edge is lower than the probability of choosing one non edge, and the number of edges in the training set is much higher than the number in the test set. Then we have $tpe = 0.015$ and finally $tpe = 0.03$, where the probability of selecting one edge in the test set is higher than the probability of choosing one non edge, and the test set has a bigger number of positive examples. These settings follow an increasing order of complexity, starting from an under-represented case, where $tpe = 0.001$, and ending with a difficult task where the number of edges in the test set is over-represented, $tpe = 0.03$. We run 10 independent trials for each setting and model.

Edge bias sampling	Attribute	Method	AUC for link prediction Ala 2013	Ala 2017	Ten 2013	Ten 2017
$tpe = 0.001$		MULTITENSOR	0.82±0.01	0.85±0.01	0.78±0.02	0.82±0.02
	Caste	MTCOV	**0.851±0.008**	**0.87±0.01**	**0.85±0.01**	**0.83±0.02**
	Religion	MTCOV	0.82±0.02	0.85±0.02	0.83±0.02	0.83±0.01
	Age	MTCOV	0.83±0.01	0.83±0.01	0.81±0.02	0.821±0.009
	Gender	MTCOV	0.81±0.02	0.85±0.02	0.83±0.01	0.82±0.01
$tpe = 0.004$		MULTITENSOR	0.771±0.009	0.835±0.006	0.758±0.005	0.81±0.01
	Caste	MTCOV	**0.846±0.007**	**0.865±0.006**	**0.838±0.006**	**0.82±0.01**
	Religion	MTCOV	0.816±0.009	0.83±0.01	0.81±0.01	0.82±0.01
	Age	MTCOV	0.82±0.01	0.83±0.01	0.791±0.009	0.79±0.02
	Gender	MTCOV	0.790±0.007	0.83±0.02	0.81±0.01	0.82±0.02
$tpe = 0.015$		MULTITENSOR	0.62±0.01	0.73±0.01	0.68±0.01	0.76±0.01
	Caste	MTCOV	**0.806±0.008**	**0.847±0.006**	**0.811±0.007**	**0.812±0.006**
	Religion	MTCOV	0.76±0.01	0.81±0.01	0.75±0.01	0.78±0.01
	Age	MTCOV	0.68±0.01	0.77±0.02	0.68±0.02	0.74±0.03
	Gender	MTCOV	0.71±0.01	0.79±0.02	0.70±0.01	0.76±0.02
$tpe = 0.03$		MULTITENSOR	0.46±0.01	0.554±0.009	0.55±0.01	0.63±0.01
	Caste	MTCOV	**0.73±0.01**	**0.79±0.01**	**0.760±0.008**	**0.77±0.01**
	Religion	MTCOV	0.68±0.02	0.74±0.02	0.64±0.02	0.68±0.01
	Age	MTCOV	0.54±0.02	0.63±0.02	0.54±0.01	0.61±0.02
	Gender	MTCOV	0.58±0.01	0.66±0.02	0.55±0.02	0.65±0.01

Table S3. Prediction performance on real multilayer networks with attributes and bias sampling on edges. Results are averages and standard deviations over 10 independent trials of cross-validation with 80-20 splits applied to the entries of A (the whole X is given in input); best performances are in boldface. Datasets are described in the main manuscript.

S4 Single-layer networks experiments

We generate 10 independent realization with different random initializaton for each network. We run NC setting the maximum number of BP steps before aborting to 40 and maximum number of EM steps before aborting to 500 to be consistent with MTCOV. MTCOV and CESNA use the fraction match as weight between the network and attributes. Moreover, for CESNA we add a new community with all non-classified nodes, and this is done for all tests. Therefore, NC and MTCOV give the possibility to decide if considering a hard membership or a mixed one, thanks to the posterior probabilities given as output, and assigning the community with the highest probability, even though NC doesn't mention the possibility of mixed-membership in the paper. On the other hand, CESNA does not return those values, and we have to work with their output files which provide an overlapping partition of the nodes.

S4.1 Single-layer synthetic networks

In analogy with what done for multilayer synthetic networks, we test MTCOV's ability to detect communities on synthetic single-layer networks, using the approach proposed by Newman and Clauset [1], where they generate synthetic networks with known community structure embedded within them. Networks are generated using the stochastic block model [2, 3], with $N = 1000$ nodes and $C = 2$ non-overlapping communities of equal size. Edge probabilities are $p_{in} = c_{in}/n$ and $p_{out} = c_{out}/n$, for within-group and between-group edges, respectively. The difference $c_{in} - c_{out}$ measures the strength of the community structure, when c_{in} is much greater than c_{out} the communities are easy to detect from network structure alone, and it becomes harder when these two quantities are close. Discrete-valued attributes are generated on nodes, which match the true community assignments of nodes a given fraction of the times, and are instead chosen uniformly at random from the non-matching values otherwise. This allows to control the correlation between attributes and community structure and hence test the algorithm's ability to exploit the extra information of varying quality. The match between attributes and planted communities varies between 0.5 and 0.9, the higher this value the higher the extent to which node attributes help predicting edges. In Fig. S2 we show the fraction of correctly classified nodes in terms of F1-score (results in terms of Jaccard are similar) for such experiments. We notice first a clear pattern where all the methods increase their performance and reduce their variance as the difference $c_{in} - c_{out}$ gets bigger, going from a hard to an easy to detect regime. In the hard regime, where community structure weakens, both MTCOV and NC remain robust in detecting the communities for scenarios where attributes are correlated. However, MTCOV shows lower variance and has more stable results for high attribute correlations. In addition, empirically we observe that NC does not reach converge in 19% of the trials, while MTCOV only for the 13% of the times. CESNA always shows lower performances, probably penalized by the relative high number of non classified examples which we also observe experimentally.

S4.2 Single-layer real networks

The *facebook* ego-networks We consider the attributes *Birthday (B), Hometown (H)* and *Location (L)*, relationship that potentially correlate with the community partitions. In addition, they have a reasonable number of values, compared to other attributes whose proportions of missing values are too high. We take in consideration also the sum by row: since we are assuming a multinomial distribution for the attribute, we ask for a sum by row equal to 1. As first step, we combine all the 10 ego networks merging both the edges and the covariates. In this way we double check the real number of edges and the number of nodes. Moreover, we build complete design matrices for B, H and L. They are used for retrieving the largest possible number of attributes for each node. However, we decide to work with the ego networks separately, for having a clear classification of the ground truth. We build the input files using the following procedure, where we consider only the nodes having at least one edge and the attribute. We obtain 30 networks: 3 for each ego network according to the attribute B, H or L; and for each combination of ego and attribute we have different adjacency matrix A and ground truth *circles*. However, we analyse 21 networks because we have to discard three ego networks (3437, 3980, 698) due to the null number of communities or to the small number of nodes with ground truth.

American College Football We use the corrected version of the dataset [4], where among all they assign the independent teams to a unique community label rather than assigning them a single community label as in the original football dataset. In this way the number of communities given by the number of conferences is 19.

Political Blogs (polblogs) We remove the isolated vertices and self-loops. The ground-truth communities are *left/liberal, right/conservative*, so $C = 2$.

For the analysis of these networks, we run CESNA with the default parameter $\alpha = 0.5$ because their released code does not allow to perform a cross-validation procedure on the scaling parameter. The facebook networks have overlapping communities, while for the other two datasets we assume non-overlapping, according to the proposed ground-truth. For facebook we consider the 21 ego networks as different iterations. For the other two, since both MTCOV and NC are based on a EM algorithm which does not ensure to reach a global maximum, we perform 10 restarts of the algorithms with different initializations at random. Results are presented in the main manuscript.

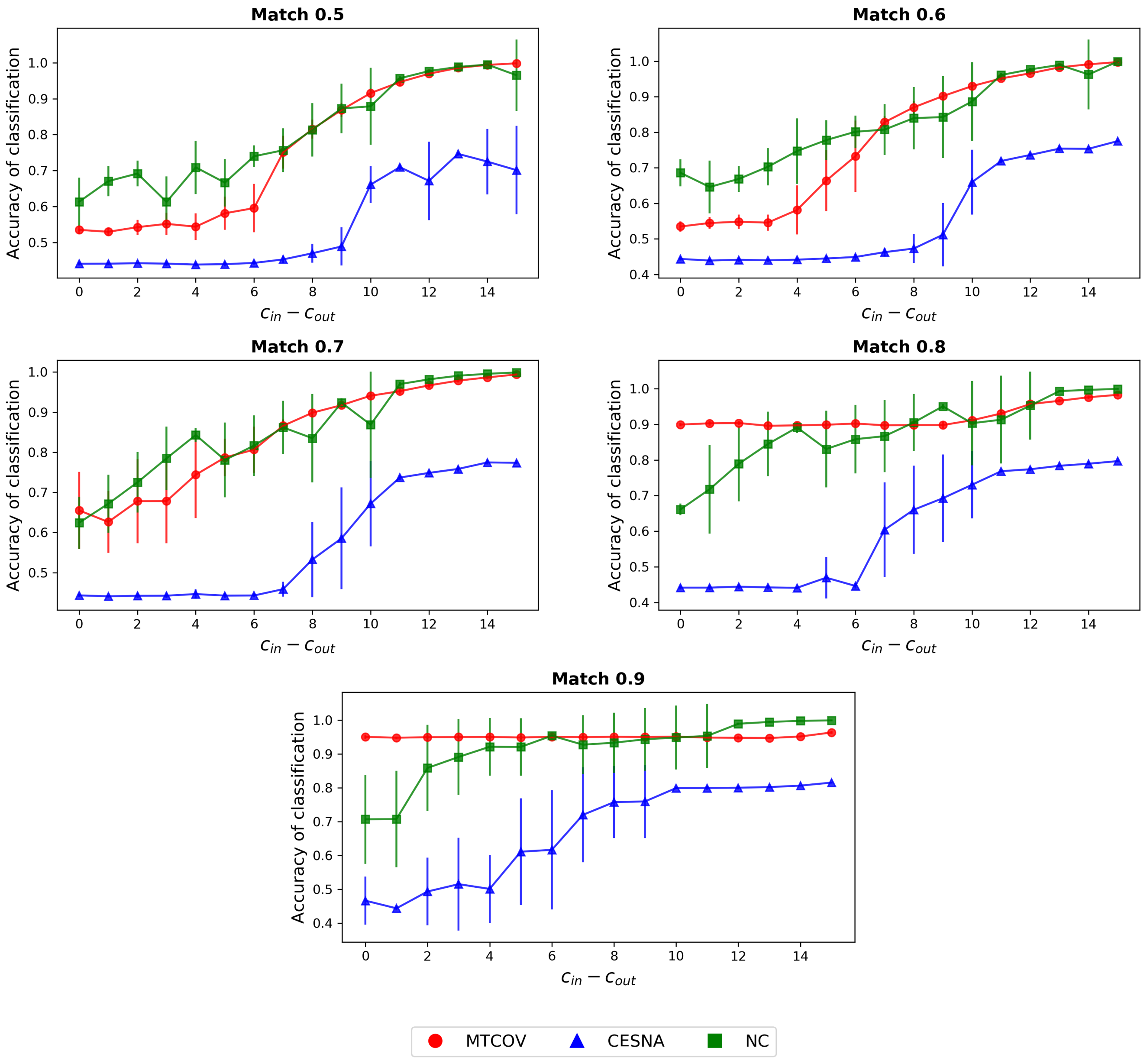

Figure S2. Accuracy of classification for synthetic single-layer networks with two communities of equal size, generated with the stochastic block model. Each plot shows results with a given match between metadata and planted communities. The results are averaged over 10 independent trials and the bars represent the standard deviation. The accuracy is measured with F1-score as similarity measure.

References

[1] Newman, M. E. & Clauset, A. Structure and inference in annotated networks. *Nat. communications* **7**, 11863 (2016).

[2] Holland, P. W., Laskey, K. B. & Leinhardt, S. Stochastic blockmodels: First steps. *Soc. networks* **5**, 109–137 (1983).

[3] Karrer, B. & Newman, M. E. Stochastic blockmodels and community structure in networks. *Phys. review E* **83**, 016107 (2011).

[4] Evans, T. S. Clique graphs and overlapping communities. *J. Stat. Mech. Theory Exp.* **2010**, 12037 (2010).
